# Discovery of a small molecule inhibitor targeting dengue virus NS5 RNA-dependent RNA polymerase

**DOI:** 10.1371/journal.pntd.0007894

**Published:** 2019-11-18

**Authors:** Hideaki Shimizu, Akatsuki Saito, Junko Mikuni, Emi E. Nakayama, Hiroo Koyama, Teruki Honma, Mikako Shirouzu, Shun-ichi Sekine, Tatsuo Shioda

**Affiliations:** 1 RIKEN Center for Biosystems Dynamics Research, Suehiro-cho, Tsurumi-ku, Yokohama, Japan; 2 Research Institute for Microbial Diseases, Osaka University, Osaka, Japan; 3 Drug Discovery Chemistry Platform Unit, RIKEN Center for Sustainable Resource Science, Hirosawa, Wako, Saitama, Japan; Oregon Health and Science University, UNITED STATES

## Abstract

Dengue is a mosquito-borne viral infection that has spread globally in recent years. Around half of the world’s population, especially in the tropics and subtropics, is at risk of infection. Every year, 50–100 million clinical cases are reported, and more than 500,000 patients develop the symptoms of severe dengue infection: dengue haemorrhagic fever and dengue shock syndrome, which threaten life in Asia and Latin America. No antiviral drug for dengue is available. The dengue virus (DENV) non-structural protein 5 (NS5), which possesses the RNA-dependent RNA polymerase (RdRp) activity and is responsible for viral replication and transcription, is an attractive target for anti-dengue drug development. In the present study, 16,240 small-molecule compounds in a fragment library were screened for their capabilities to inhibit the DENV type 2 (DENV2) RdRp activities *in vitro*. Based on *in cellulo* antiviral and cytotoxity assays, we selected the compound RK-0404678 with the EC_50_ value of 6.0 μM for DENV2. Crystallographic analyses revealed two unique binding sites for RK-0404678 within the RdRp, which are conserved in flavivirus NS5 proteins. No resistant viruses emerged after nine rounds of serial passage of DENV2 in the presence of RK-0404678, suggesting the high genetic barrier of this compound to the emergence of a resistant virus. Collectively, RK-0404678 and its binding sites provide a new framework for antiviral drug development.

## Author summary

Dengue is a mosquito-borne infection caused by dengue viruses (DENV), and is currently a major public health concern worldwide. No antiviral drug for dengue is available. To develop a potent inhibitor of the DENV NS5 RNA-dependent RNA polymerase (RdRp), we performed a high-throughput screening of a fragment library. We identified RK-0404678 as a potent inhibitor of the DENV RdRp. Interestingly, we found that RK-0404678 binds to two distinct sites in the DENV RdRp domains. Site 1 lies in the thumb domain, which is distant from the active site, and Site 2 is located in the active site of the RdRp domain. RK-0404678 binding to Site 2 induces a conformational change around the Tyr607 residue. These are unique features among the known RdRp inhibitors. In addition, our adaptation experiment demonstrated that this compound imposed a high genetic barrier to the emergence of an RK-0404678-resistant virus. These characteristics of RK-0404678 suggest that this inhibitor is a promising lead compound for the development of anti-dengue therapeutics.

## Introduction

Dengue is a mosquito-borne viral infection that has spread globally in recent years. Around half of the world’s population, especially in the tropics and subtropics, is at risk of infection. Every year, 50–100 million clinical cases are reported, and more than 500,000 patients develop the symptoms of severe dengue infection: dengue haemorrhagic fever (DHF) and dengue shock syndrome (DSS) [1], which threaten life in Asia and Latin America. The causative agent of dengue is the dengue virus (DENV), which is classified into four distinct serotypes (DENV1, 2, 3, and 4), sharing 70–80% identities in the ORF amino-acid sequences [2]. The cross infection of a patient who already has immunity against one serotype with another serotype increases the risk of severe dengue [3]. Currently, there is no specific antiviral treatment for dengue.

DENV belongs to the *Flaviviridae* family of positive-strand RNA viruses, including human pathogens such as Zika virus (ZIKV), West Nile virus (WNV), yellow fever virus (YFV), Japanese encephalitis virus (JEV), and hepatitis C virus (HCV) [4]. The DENV genome encodes three structural proteins (capsid protein C, membrane protein M, and envelope protein E) and seven non-structural proteins (NS1, NS2a, NS2b, NS3, NS4a, NS4b, and NS5) [4, 5]. Non-structural protein 5 (NS5) is the largest viral protein, comprising the N-terminal methyltransferase (MTase) domain and the C-terminal RNA-dependent RNA polymerase (RdRp) domain. The amino-acid sequence identities of NS5 are around 70% among the four DENV serotypes [6]. The MTase domain catalyzes the methylation of the RNA 5´-cap structure, to generate N7-methyl-guanosine and 2´-O-methyl adenosine (GpppA-RNA → m^7^GpppAm-RNA) [6]. The RdRp domain is essential for both positive- and negative-strand RNA syntheses during replication [7]. Due to the absence of a similar protein in humans, RdRp is a potential target for the development of specific antiviral inhibitors against DENV [8, 9].

The structure of RdRp is often compared to a right hand, and is composed of three subdomains: thumb, fingers, and palm. The thumb subdomain contains a so-called priming loop, which extends into a double-stranded RNA binding site, and is hypothesized to undergo a conformational change during the *de novo* initiation of RNA synthesis [10]. Many inhibitors against DENV RdRp reportedly bind near the priming loop [11–13]. Several inhibitors against HCV RdRp bind to the “allosteric sites” on the thumb subdomain of the RdRp [14]. The DENV RdRp was also predicted to possess allosteric sites on the thumb subdomain [8], but no inhibitors targeting these sites have been reported so far.

To develop a potent inhibitor of DENV NS5 RdRp, we performed a high-throughput screening (HTS) of a fragment library, and obtained a hit compound (termed RK-0404678). The EC_50_ values for RK-0404678 were 6.0–31.9 μM against DENV1-4, and we also determined the crystal structures of the DENV NS5 RdRp domain bound with RK-0404678. Interestingly, two distinct binding sites of RK-0404678 were observed, and mutagenesis studies suggested that both binding sites contribute to the inhibition of the RdRp activity by RK-0404678. Serial passages of DENV2 in the presence of this compound did not yield RK-0404678-resistant viruses, suggesting the high genetic barrier of this compound to the emergence of a resistant virus. Thus, this compound and its binding sites provide a new foundation for the further development of effective anti-dengue drugs.

## Results

### Identification of RK-0404678

To identify chemical compounds that specifically bind to the RdRp pockets and inhibit its activity, a fragment library was screened using the purified DENV2 NS5 RdRp protein. The assay is based on the RdRp-dependent synthesis of poly-G RNA, from the template poly-C RNA and the substrate GTP (RdRp assay). The double-stranded poly-G:C RNA thus produced was quantitated by measuring the emission from the fluorescent reagent Picogreen (Molecular Probes). The fluorescence intensity was normalized by the control intensities obtained in the presence of GTP (100%, high control) and in the absence of GTP (0%, low control). The RdRp assay conditions were optimized by examining the MnCl_2_ concentrations, pH, temperature, protein concentrations, and reaction time (S1A–S1D Fig).

The first screening was performed for 16,240 fragment compounds at the concentration of 100 μM (S2A Fig). We selected the compounds that showed more than 40% inhibition of the RdRp activity. The selected compounds were reexamined in triplicate to assess the reproducibility, and thus 13 fragment compounds were selected (S2B Fig).

**Fig 1:**
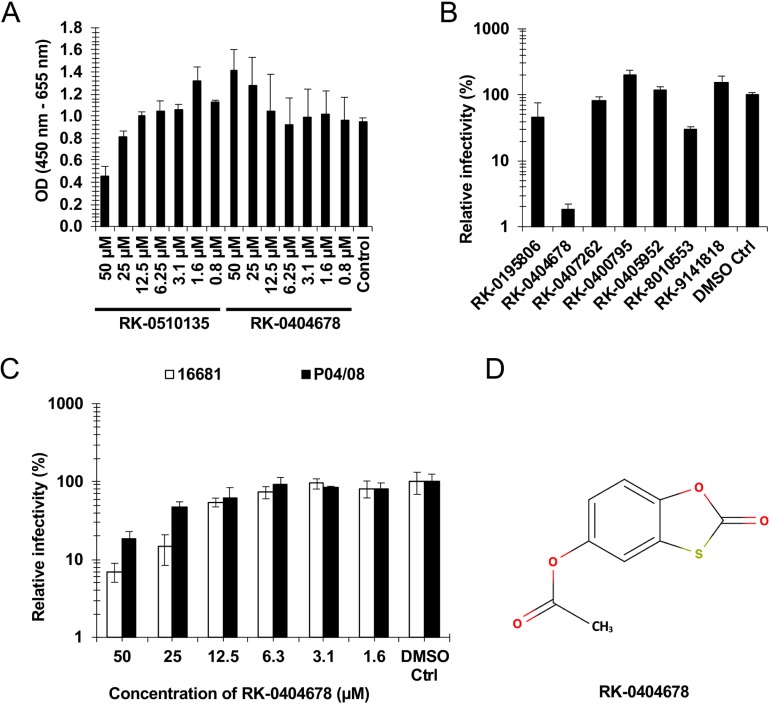
Screening of the fragment library. A. Vero cells were cultured in the presence of compounds for two days. The cytotoxicity of each compound was evaluated with the WST-1 assay reagent. The absorbance of samples was measured using a microplate reader at 450 nm. The reference wavelength was 655 nm. The results shown are the mean and standard deviation of triplicate measurements. B. Vero cells were infected with the DENV2 16681 strain in the presence of compounds. The antiviral effect of each compound was evaluated by measuring the viral RNA in the culture supernatant at 72 hours after infection. The drug sensitivity is displayed as the relative infectivity normalized to control cells without drug treatment. The results shown are the mean and standard deviation of triplicate measurements. C. Vero cells were infected with either the DENV-2 16681 or P04/08 strain in the presence of RK-0404678. The antiviral effect of each compound was evaluated by measuring the viral RNA in the culture supernatant at 72 hours after infection. The drug sensitivity is displayed as the relative infectivity normalized to control cells without drug treatment. The results shown are the mean and standard deviation of triplicate measurements. D. Chemical structure of RK-0404678.

To assess the efficacies of these compounds in cultured cells, we first tested their cytotoxicity in Vero cells, using a WST-1 assay. While several compounds, such as RK-0510135, showed cytotoxicity at high concentrations (Fig 1A), RK-0404678 showed marginal cytotoxicity even at the highest concentration (50 μM). We next tested the antiviral activity of 7 compounds at the highest concentration at which they did not show cytotoxicity. The antiviral effect of each compound was evaluated by measuring the viral RNA in the culture supernatant at 72 hours after infection. RK-0404678 suppressed the replication of the DENV2 16681 strain in up to 2–3% of untreated cells (Fig 1B). The EC_50_ value of the antiviral activity was estimated to be 6.0 μM (Table 1). We also observed a comparable effect of RK-0404678 on the different DENV2 P04/08 strain (Fig 1C), suggesting that the effect is not specific to the DENV2 16681 strain. The time course experiment demonstrated that RK-0404678 exhibited antiviral activity against the DENV2 strains at 48 and 72 hours after infection, with a milder effect at 24 hours after infection (S3 Fig).

**Table 1:** Inhibition of the RdRp activity by RK-0404678 for the full-length DENV1-4 NS5 proteins (determined as IC_50_) and inhibition of the viral replication of DENV1-4 by RK-0404678 (determined as EC_50_).

	DENV1	DENV2	DENV3	DENV4
IC_50_ (μM) Polymerase assay	46.2 ± 2.8	201 ± 4.9	287 ± 11	445 ± 23
EC_50_ (μM) Cell-based assay	29.5 ± 4.2	6.0 ± 0.30	29.4 ± 1.8	31.9 ± 2.8

The results shown are the mean and standard deviation of quadruplicate measurements for IC_50_ values in a single experiment and triplicate measurements for EC_50_ values. For EC_50_ values, one representative result of three independent experiments is shown. The IC_50_ and EC_50_ values were compared by Extra Sum-of-Squares F test in PRISM version 6 (GraphPad Software, Inc.). The p-values for differences in IC_50_ values were p < 0.0001 for all combinations of the four serotypes. The p-values for differences in EC_50_ values are as follows: p < 0.0001 for DENV1–DENV2, DENV2–DENV3, and DENV2–DENV4; p = 0.135 for DENV1–DENV3; p = 0.131 for DENV1–DENV4; p = 0.294 for DENV3–DENV4.

Accordingly, we selected the candidate inhibitor compound RK-0404678 (Fig 1D). It inhibited the RdRp activities with IC_50_ values of 46.2–445 μM for the NS5 proteins from DENV1–4, and exhibited antiviral activities with EC_50_ values of 6.0–31.9 μM for the DENV1–4 infections (Table 1). DENV2 was the most sensitive to RK-0404678 in cultured cells.

### The binding modes of RK-0404678 to RdRp

**Fig 2:**
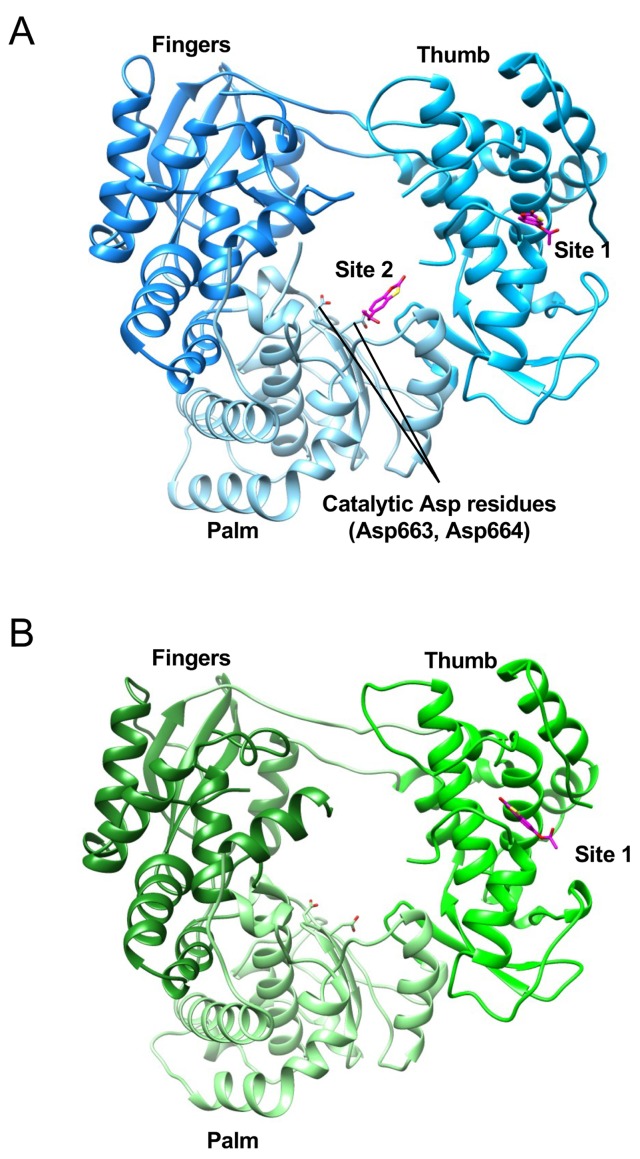
Crystal structures of the DENV2 and DENV3 RdRp domains in complex with RK-0404678. A-B. Overall structures of the DENV2 (A) and DENV3 (B) RdRps, shown in ribbon representations. The bound RK-0404678 molecules are depicted by magenta stick models.

In order to understand the inhibitory mechanism of RK-0404678, the crystal structures of the NS5 RdRp domains from DENV2 and DENV3 were determined in the complex form with RK-0404678 at 2.43 and 1.97 Å resolutions, respectively (Fig 2A and 2B). The DENV2 RdRp structure revealed two RK-0404678 molecules bound to two distinct sites (Sites 1 and 2), while the DENV3 RdRp structure showed only one molecule of the compound bound to Site 1.

**Fig 3:**
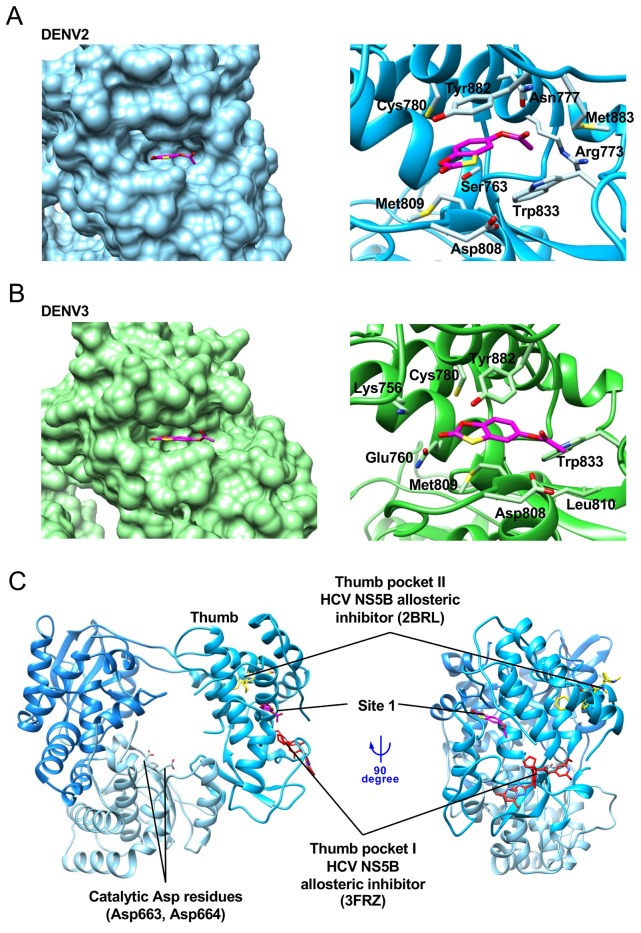
Details of the interaction between the DENV RdRps and RK-0404678 in Site 1. A. Surface (left) and ribbon-stick (right) representations of the interaction between the DENV2 RdRp and RK-0404678 in Site 1. RK-0404678 is represented by a magenta stick model. B. Surface (left) and ribbon-stick (right) representations of the interaction between the DENV3 RdRp and RK-0404678 in Site 1. C. Binding sites of allosteric inhibitors in the thumb subdomain. Allosteric inhibitors for HCV NS5 RdRp, represented by red (PDB ID: 3DRZ) and yellow (PDB ID: 2BRL) stick models, are superimposed on the structure of the RK-0404678-bound form of the DENV2 RdRp.

Site 1 is a pocket formed on the thumb subdomain, and is ~30 Å distant from the RdRp active site. This pocket is narrow and concave (Fig 3A and 3B, left), and RK-0404678 forms multiple contacts with its amino-acid residues. In the DENV2 RdRp structure, the benzoxathiole ring of RK-0404678 is surrounded by the side chains of Cys780, Tyr882, Met809, Ser763, and Asp808, while the acetate group interacts with the side chains of Trp833, Arg773, Met883, and Asn777 (Fig 3A, right). In the DENV3 RdRp structure, RK-0404678 is bound to the same pocket, but its location is slightly shifted as compared with that in the DENV2 RdRp structure. The benzoxathiole ring of RK-0404678 contacts the side chains of Cys780, Tyr882, Met809, Glu760, and Lys756, while the acetate group interacts with the side chains of Trp833, Asp808, and Leu810 (Fig 3B, right).

**Fig 4:**
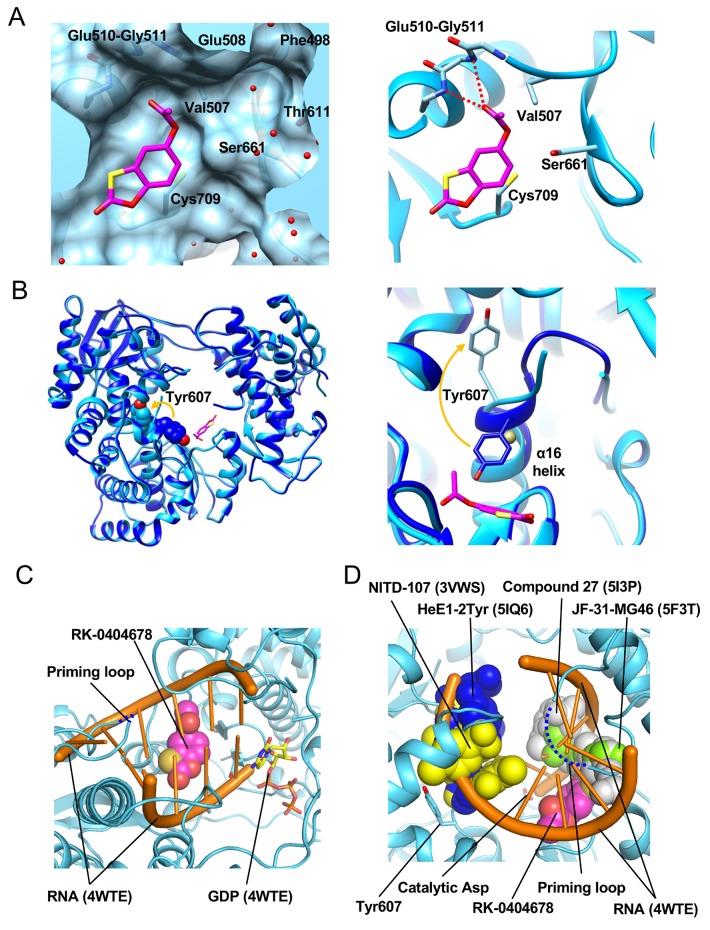
Details of the interaction between the DENV2 RdRp and RK-0404678 in Site 2. A. Surface (left) and ribbon-stick (right) representations of the interaction between the DENV2 RdRp and RK-0404678 in Site 2. RK-0404678 is represented by a magenta stick model. The water molecules and hydrogen bonds are represented by red spheres and dotted lines. B. Structural comparison of Tyr607 between the RK-0404678-bound (light blue) and apo (blue) forms. Overall structures (left) and close-up view (right). The side chains of Tyr607 are represented by CPK (left) and stick models (right), and the cobalt atom is represented by a gold sphere. C. Site 2 in the active site of the RdRp. The RK-0404678 molecule (magenta) is represented by a CPK model. The RNA strands (orange) and GDP (stick model) of the HCV RdRp structure (PDB ID: 4WTE) are superimposed on the DENV2 RdRp structure. The priming loop, which is not visible in the DENV2 RdRp structure, is represented by a blue dashed line. D. Overlay of the previously reported DENV inhibitors in the active site of the RdRp. The RK-0404678 molecule (magenta), the previously reported inhibitors (PDB IDs: 5I3P (grey), 5F3T (light green), 3VWS (blue), 5IQ6 (yellow)), and the RNA strands and GDP (PDB ID: 4WTE, orange) are superimposed on the DENV2 RdRp structure. The priming loop is represented by a blue dashed line.

The DENV2 RdRp structure revealed the second binding site (Site 2) for RK-0404678. This site is within the RNA-binding cleft, close to the catalytic Asp residues on the palm subdomain (Fig 2A). The benzoxathiole ring of RK-0404678 interacts with the side chains of Val507, Ser661, and Cys709 and the main chains of Glu510-Gly511 (Fig 4A). A comparison of the DENV2 RdRp structures with and without the bound RK-0404678 revealed that the compound binding induces a local conformational change in the RdRp (Fig 4B). While Tyr607 on the α16 helix faces the catalytic center in the absence of the compound, it is flipped in the opposite direction upon compound binding. This change affects the structures of the α16-helix and its flanking loop, which are disordered in the presence of the compound. A cobalt ion included in the crystallization conditions occupies the space left after the flipping of the Tyr607 side chain. In the DENV3 RdRp structure, no electron density corresponding to RK-0404678 was observed at Site 2, and Tyr607 is in the unflipped conformation.

The DENV2 RdRp structure was compared with the structure of the HCV RdRp bound with RNA and GDP (PDB: 4WTE) by superimposition [15]. This comparison revealed that RK-0404678 at Site 2 clashes with both the template and non-template RNA strands, but does not overlap with the GDP molecule. Therefore, RK-0404678 binding to Site 2 is incompatible with RNA binding and RNA synthesis (Fig 4C). The crystal structures of DENV RdRp bound with several other inhibitors have also been reported. NITD-107 [16] and HeE1-2Tyr [17] bind to the fingers subdomain, and are apart from Site 2. Other inhibitors, such as JF-31-MG46 [13] and compound 27 [11], partially overlap with RK-0404678 at Site 2 (Fig 4D).

### Mutations of the cysteine residues in DENV2

The crystal structure of DENV2 RdRp revealed that cysteine residues commonly contact the benzoxathiole ring of RK-0404678 in Sites 1 and 2 (Cys780 and Cys709, respectively) (S4A–S4D Fig). These Cys residues are highly conserved in DENV1–4 (S5 Fig). To assess the significance of Sites 1 and 2 for the RdRp inhibition by RK-0404678, we created full-length DENV2 NS5 proteins with the mutations of Cys780 and Cys709 to either Ala or Gln, and tested their activities in the absence or presence of RK-0404678. These mutant proteins retained RdRp activities ranging from 31.8 to 87.7%, as compared with the WT protein (Table 2). The C780A and C780Q mutant proteins exhibited similar IC_50_ values, as compared with the WT protein. In contrast, the C709A and C709Q mutant proteins exhibited 2.1- and 1.8-fold increased IC_50_ values over the WT protein. These results suggested the significance of Site 2 (Table 2).

**Table 2:** Inhibition of the RdRp activity by RK-0404678 for the full-length DENV1 and DENV2 NS5 mutant proteins.

	Activity compared to WT (%)	IC_50_ (μM)
DENV1 NS5		
WT	100	46.2 ± 2.7
Site 1		
C779A	82.2	65.5 ± 4.7 **
C779Q	94.9	137 ± 5.8 ****
Site 2		
C708A	46.4	93.8 ± 7.0 ****
C708Q	48.6	170 ± 8.3 ****
Site 1 & Site 2		
C779A/C708A	24.8	163 ± 16 ****
C779Q/C708Q	29.6	181 ± 13 ****
DENV2 NS5		
WT	100	201 ± 4.9
Site 1		
C780A	31.8	206 ± 6.1 ^§^
C780Q	87.7	227 ± 6.9 ****
Site 2		
C709A	54.7	427 ± 27 ****
C709Q	53.7	368 ± 12 ****

The results shown are the mean and standard deviation of quadruplicate measurements in a single experiment. The IC_50_ values were compared by Extra Sum-of-Squares F test in PRISM version 6 (GraphPad Software, Inc.).

**p < 0.01

***p < 0.001

****p < 0.0001, and

^§^p = 0.01, compared with WT.

We next investigated the efficacy of the inhibitor in cultured cells. We generated DENV2 NS5 mutants harboring mutations in the amino acid residues interacting with RK-0404678, to test the relevance of the antiviral effect of RK-0404678 to its binding sites in the NS5 protein. We constructed the C709A, C709Q, C780A, and C780Q mutants of DENV2 NS5 to perturb the interactions with RK-0404678. Although we tried to rescue these viruses using the CPER technique, both the C709A and C709Q mutants failed to replicate in our system (S6 Fig). This result suggested that the cysteine residue at position 709 is critical for the enzymatic activity of NS5, and a mutation at this position abolishes its activity. In contrast, we succeeded in rescuing the C780A and C780Q viruses. These viruses showed similar replication capabilities as compared with the WT virus, suggesting that the amino acid substitutions at this position are tolerable for viral replication. We then examined the sensitivity of these viruses to RK-0404678. The calculated EC_50_ values of the WT, C780A, and C780Q viruses were 7.1, 3.3, and 12.8 μM, respectively (Table 3), and there is no big differences in sensitivities to RK-0404678 between the WT and mutant viruses. Accordingly, Site 1 does not appear to play a significant role in the inhibition of the RdRp activity in DENV2.

**Table 3:** Antiviral activity of RK-0404678 for DENV2 mutants.

	EC_50_ (μM)
WT (recombinant)	7.1 ± 0.67
Site 1	
C780A	3.3 ± 1.1 **
C780Q	12.8 ± 2.2 ^§^
Site 2	
C709A	not determined
C709Q	not determined

The results shown are the mean and standard deviation of triplicate measurements. One representative result of three independent experiments is shown. The EC_50_ values were compared by Extra Sum-of-Squares F test in PRISM version 6 (GraphPad Software, Inc.).

**p < 0.01 and

^§^p = 0.0445, compared with WT.

### Mutations of cysteine residues in DENV1

We also examined the significance of Sites 1 and 2 for the inhibition of the DENV1 RdRp activity by RK-0404678, as this compound showed the highest inhibitory effect against the DENV1 RdRp in the *in vitro* enzyme assay. Cys779 and Cys708 of the DENV1 NS5 correspond to Cys780 and Cys709 of the DENV2/DENV3 NS5s, respectively (S7 Fig). We mutated Cys779 and Cys708 to either Ala or Gln, and examined the activities of these mutants in the absence or presence of RK-0404678. The RdRp activities of these DENV1 NS5 mutant proteins ranged from 24.8 to 94.9%, as compared with the WT protein (Table 2). The C779A mutant protein showed a slightly increased IC_50_ value, and the C779Q mutant protein showed a 3.0-fold increased IC_50_ value, as compared with the WT protein. The C708A and C708Q mutant proteins exhibited 2.0- and 3.7-fold increased IC_50_ values, respectively. Thus, the C779Q and C708Q mutations, in which Cys is replaced by the bulkier Gln to block the compound binding site, exhibited higher IC_50_ values than the C779A and C708A mutations. Moreover, the double mutant C779A/C708A and C779Q/C708Q proteins exhibited 3.5- and 3.9-fold increased IC_50_ values, respectively (Table 2). These results suggest the involvement of both Sites 1 and 2 in the RK-0404678-mediated DENV1 NS5 inhibition, while the Site 2 binding by RK-0404678 is slightly more effective than the Site 1 binding.

### High genetic barrier of RK-0404678 to the emergence of resistant viruses

The identification of drug-resistant viruses will provide information about not only how DENV evades inhibition but also which viral components form the binding interface to the drug. A previous study demonstrated that the adaptation of the DENV replicon in the presence of antiviral compounds targeting NS5 led to the emergence of drug-resistant clones harboring mutations in the NS5 gene [11]. To investigate if an RK-0404678-resistant virus could emerge, we performed an adaptation test of the DENV2 16681 virus to RK-0404678. The virus was passaged nine times in Vero cells cultured in medium containing 50 μM RK-0404678. The passaged virus, termed P9, did not gain obvious resistance to RK-0404678 (S8 Fig), and the calculated EC_50_ value was 1.0 μM. This result suggests that RK-0404678 possesses a high genetic barrier to the emergence of resistant viruses.

## Discussion

Through the HTS approach and cell-based assays, RK-0404678 was identified as a novel inhibitor for the DENV RdRp. Interestingly, RK-0404678 was revealed to have two distinct binding sites in the DENV RdRp domains. Site 1 lies in the thumb domain, which is distant from the active site, and Site 2 is located in the active site of the RdRp domain. RK-0404678 binding to Site 2 induces a conformational change around the Tyr607 residue. These are unique features among the known RdRp inhibitors. In addition, our adaptation experiment demonstrated that this compound imposed a high genetic barrier to the emergence of an RK-0404678-resistant virus. These characteristics of RK-0404678 suggest that this inhibitor is a promising lead compound for the development of anti-dengue therapeutics.

To assess the chemical tractability of RK-0404678 for further development, the calculation of the Lipinsky rule of five properties [18] and the machine learning prediction of metabolic stability were performed. In terms of the rule of five, the criteria (log P < 5, molecular weight < 500, number of H-bonding acceptors < 10, number of H-bonding donors < 5) are used for the judgment of drugabbility. We calculated the properties by Pipeline Pilot [19], and the results met the above-mentioned criteria (log P: 2.31, molecular weight: 210.2, number of H-bonding acceptors: 5, number of H-bonding donors: 0). In addition, the prediction model of human microsomal stability developed by Esaki et al. [20] categorized RK-0404678 as “Stable (CLint < 20 μL/min/mg)”. Based on these preliminary assessments, RK-0404678 appears to be a promising starting point for future drug discovery.

As mentioned above, cysteine residues are the common structural features of Sites 1 and 2, and both of the cysteine residues are involved in direct contacts with RK-0404678 (S4A–S4C Fig). Site 2 is a common inhibitory site between DENV1 and DENV2, while Site 1 also contributes to the inhibition of DENV1 NS5. The results of the Cys to Ala mutations, showing increased IC_50_ values, suggested that the SH group of the Cys residues contributes to the RK-0404678 binding. Interestingly, the corresponding Cys residues are conserved across the DENV (S5 Fig), ZIKV, JEV, and WNV NS5 proteins [21, 22], suggesting that RK-0404678 could also bind to these flavivirus RdRps.

Our crystal structures revealed that Site1 is located in a characteristic narrow pocket of the thumb domain. This pocket is conserved in the flavivirus RdRp structures, and has been proposed to be a potentially druggable allosteric inhibition site [8]. However, no inhibitor for this pocket has been reported, until this study. In *Flaviviridae*, HCV NS5B possesses two allosteric sites in the thumb domain (termed thumb pockets I and II) [14] (Fig 3C). The most effective compounds for thumb pockets I and II inhibited the RdRp activity of recombinant NS5B with IC_50_ values in the low nM range and the HCV RNA replication with EC_50_ values in the sub-μM range [14]. However, these pockets are not conserved in the RdRps from flaviviruses, including DENV. In contrast, Site 1 is not conserved in HCV RdRp, but is a unique feature of the flaviviruses (Fig 3C). Although contribution of Site 1 differs between DENV1 and DENV2, the current structure of DENV RdRp bound with RK-0404678 at Site 1 provides a new framework for the future development of effective RdRp inhibitors against flaviviruses.

As shown in Fig 4C, Site 2 is located within the RNA binding site, suggesting that RK-0404678 binding at Site 2 directly hinders the RNA binding. Additionally, the RK-0404678 binding to Site 2 causes the flipping of the Tyr607 side chain (Fig 4B), and this change also might contribute to the inhibition by RK-0404678. Tyr607 is located in the vicinity of the binding sites of several allosteric inhibitors [11–13, 17] (Fig 4D). RK-0404678 is a small molecule with a molecular mass of 210.2 Da. The space around Site 2 is wide, and another pocket composed of Phe498, Glu508, Thr611, and Ser661 exists beyond the acetate group of RK-0404678 (Fig 4A). Extending the compound into this pocket may enhance the inhibitory activity.

A major concern in the development of antiviral compounds is the emergence of drug-resistant viruses. In the influenza virus field, an oseltamivir-resistant virus was disseminated to the human population [23, 24]. Just one amino acid substitution in the viral neuraminidase was sufficient to generate drug resistance. Resistance to oseltamivir is caused primarily by mutations in either the active-site residues or those that change the shape of the catalytic site [25]. Resistant dengue viruses emerged after the adaptation of the viruses to the presence of the nucleoside analog 2′-C-methyl-7-deaza-adenosine (7-DMA) [26]. The resistant viruses harbored mutations in NS5. Therefore, it is important to monitor for the emergence of resistant viruses during anti-viral drug development. In this study, nine rounds of serial passage did not yield RK-0404678-resistant viruses. In a previous evaluation of NS5-targeted compounds, the adaptation of the DENV2 replicon to generate resistant clones occurred within five weeks [11]. This finding, along with the genetic stability of NS5 due to its pivotal role in viral replication, suggests that the development of resistance to compounds targeting NS5 is a difficult process. In future studies, we will determine whether extended passages of DENV in the presence of RK-0404678 generate RK-0404678-resistant viruses.

Darunavir, an HIV-1 inhibitor targeting the viral protease [27], shows high antiviral potency and a strong genetic barrier. These characteristics are explained by its binding property to the target molecule: Darunavir binds to the protease active site through interactions with the mainchain residues (Asp29 and Asp30) [28]. Our structural analyses demonstrated that RK-0404678 binds to Site 2 of the DENV2 RdRp through interactions with the mainchains of Glu510-Gly511 (Fig 4A). In addition to the genetic stability of NS5, as discussed above, this characteristic of RK-0404678 might partially explain the high genetic barrier for the RK-0404678-resistant virus development, and highlights the potential of this compound and Site 2 for further drug development.

As shown in Table 1, DENV2 was the most sensitive to RK-0404678 in cultured cells. In the *in vitro* enzyme assay, however, DENV1 showed the lowest IC_50_ value. The reason of this discrepancy is not clear. One possibility is that RK-0404678 has other targets than NS5 *in cellulo*, and it eventually has more effect on DENV2 RdRp than on DENV1 RdRp. Another possibility is that that RK-0404678 might be metabolized to another form *in cellulo*, and the derivative could have more effect on DENV2 RdRp than on DENV1 RdRp.

In summary, using biochemical and virological approaches, we identified RK-0404678 as a potent inhibitor of the DENV RdRp. Our structural studies further demonstrated that RK-0404678 binds to two unique sites. This structural and functional information will be useful for improving the compound for the future development of new druggable molecules, not only for the DENV RdRps but also for the other flavivirus RdRps.

## Methods

### Protein expression and purification

#### DENV2 RdRp for fragment screening and crystallization

The DNA fragment encoding the DENV2 RdRp domain (a.a. 251–896) (AF038403) was cloned into the expression vector pCR2.1 TOPO (Life Technologies), with N-terminal His- and Flag-tags and a TEV protease cleavage site. We also added a sequence composed of glycine and serine residues (GSSGSSG) at the N-terminus, as a linker between the cleavage site and the body of RdRp. The sequences of the primers used in this study are shown in S2 Table. The protein was produced by the *Escherichia coli* cell-free protein synthesis method [29]. Briefly, transcription and translation reactions were performed in a single tube at 25°C for 4 hours in the presence of the template plasmid DNA, T7 RNA polymerase, S30 extract, NTPs, amino acids, and ZnCl_2_. After the reaction, the reaction mixture was centrifuged at 30,000 *g* at 4°C for 30–60 min, and the supernatant was loaded onto a HisTrap column, equilibrated with 50 mM Tris-HCl buffer (pH 7.0), containing 500 mM NaCl, 20 mM imidazole, 10% glycerol, and 20 μM ZnCl_2_. After washing the column with the buffer, the His-tagged proteins were eluted with 50 mM Tris-HCl buffer (pH 7.0), containing 500 mM NaCl, 500 mM imidazole, 10% glycerol, and 20 μM ZnCl_2_. The N-terminal tags were cleaved by dialyzing the eluted protein against 20 mM Tris-HCl buffer (pH 7.0), containing 500 mM NaCl, 20 mM imidazole, 10% glycerol, 20 μM ZnCl_2_, 5 mM TCEP or 2-mercaptoethanol, and TEV protease [30], at 4°C overnight. The uncleaved protein was removed by passing the sample through the HisTrap column. The flow-through fractions were concentrated and loaded on a HiLoad 16/60 Superdex 200 column, equilibrated with 20 mM HEPES-NaOH buffer (pH 7.0–7.5), containing 500 mM NaCl, 10% glycerol, and 5 mM TCEP or 2-mercaptoethanol. The purified proteins were concentrated to 10–20 mg/ml. The final protein samples are >95% pure, as judged by SDS-PAGE analyses (S10 Fig).

#### NS5 proteins for IC_50_ measurements

DNA fragments encoding the full-length NS5 proteins, comprising the N-terminal MTase and the C-terminal RdRp, from DENV1, DENV3, and DENV4 were synthesized (Eurofins Genomics), and cloned into the expression vector pCR2.1 TOPO (Life Technologies) with N-terminal His- and SUMO-tags, and a SUMO protease cleavage site. The expression vector pET Duet-1 (Novagen), which contains a DNA fragment encoding the full-length DENV2 NS5 with an N-terminal His-tag and a TEV protease cleavage site, was purchased from GenScript. These amino-acid sequences, the synthesized ORF DNA sequences, and the sequences of the primers used in this study are shown in S7 Fig, S9 Fig, and S2 Table. The NS5 proteins were expressed in the *E*. *coli* strain KRX, cultured in terrific broth medium. When the culture reached an OD_600_ of 0.4–0.6, the protein expression was induced by adding 0.5 mM IPTG and 0.1% rhamnose, and the culture was continued for 1–2 days at 18°C. The protein purifications were performed as described above, except that SUMO protease was used instead of TEV protease when appropriate. The final protein samples are >95% pure, as judged by SDS-PAGE analyses (S10 Fig).

#### DENV3 RdRp domain for crystallography

The DNA fragment encoding the DENV3 RdRp domain (a.a. 250–896) was cloned into the expression vector pCR2.1 TOPO with N-terminal His- and SUMO-tags, and a SUMO protease cleavage site. The sequences of the primers used in this study are shown in S2 Table. The DENV3 RdRp domain was expressed in the *E*. *coli* strain KRX, and purified as described above. The final protein samples are >95% pure, as judged by the SDS-PAGE analyses (S10 Fig).

### Fragment library

We constructed a fragment library including 16,240 fragment-sized compounds ranging from 100 Da to 347 Da, and used it for the screening described in this article. The fragment-sized compounds were selected from commercially available compound databases, based on both ligand-based and structure-based approaches. In terms of the ligand-based selection, we first extracted 1,116 assay systems with more than 30 compound assay results from CheMBL [31], as of 2013. Using 163,789 compounds assayed by the 1,116 systems, we performed fragmentation analyses by every rotatable bond with every combination. Among the components of the fragment pool, 37,810 fragments that appeared >3% or >10 times in each assay system were selected. From the PDB as of 2013, all small ligands in all complex crystal structures were extracted and fragmented by every rotatable bond with every combination. Next, the binding energy values of the obtained fragments were predicted by the MM-GBVI [32] method, using the modeling software MOE 2013 (CCG-Chemical Computing Group Inc., Montreal, Quebec, Canada). From the PDB-derived 1,773,685 fragments, 23,105 fragments satisfying the criteria (1) MM-GBVI <-30 kcal/mol or (2) MM-GBVI/the number of non-hydrogen atoms <-2 kcal/mol were selected as important fragments for protein-ligand interactions. Using the 37,810 (ligand-based selection) and 23,105 (structure-based selection) fragments as queries, we searched commercially available databases, and found 24,868 commercially available fragments. Considering the rankings, prices, and delivery dates of the fragments, we purchased 16,240 fragment-sized compounds, and prepared their DMSO solution samples for the subsequent screening.

### (2-Oxo-1, 3-benzoxathiol-5-yl) acetate (RK-0404678)

RK-0404678 was purchased from Vitas-M Chemical Limited (Causeway Bay, Hong Kong) through the Japanese agent Namiki Shoji Co., Ltd. (Tokyo, Japan). The supplier ID is STK282946. We measured the purity of the purchased lot by LC-MS. LC-MS (ESI): m/z 211 (M + H+). Retention Time: 1.32 min. Purity: >96%.

### Fragment library screening

The fragment library was screened by assessing each compound’s inhibitory effect on the RdRp assay. The DENV2 RdRp protein was used for this screening. The enzymatic reactions were performed within 96-well black plates by using an automated system (Biomek FX and ORCA robotic system, Beckman Coulter). Each reaction was performed in an 8 μl reaction. A 2 μl aliquot of 4 μM DENV2 RdRp, dissolved in a reaction buffer containing 40 mM Tris-HCl (pH 7.4), 23.8 mM NaCl, 15% glycerol, 10 mM MnCl_2_, and 14 mM 2-mercaptoethanol, was mixed with 2 μl of 400 μM compound dissolved in 4% DMSO, and the mixture was incubated at 37°C for 60 min. Subsequently, 2 μl of 250 nM poly-C (SIGMA) dissolved in water was added, and the mixture was incubated at 37°C for 60 min. The reactions were initiated by adding 2 μl of 4 mM GTP dissolved in the reaction buffer to the mixture. The final concentrations of the reaction components are 1 μM RdRp, 62.5 nM poly-C, 1mM GTP, 100 μM compound, 1% DMSO, 20 mM Tris-HCl (pH 7.4), 11.9 mM NaCl, 7.5% glycerol, 5 mM MnCl_2_, and 7 mM 2-mercaptoethanol. After incubating at 37°C for 60 min, 85 μl of PicoGreen Quantitation Reagent (Molecular Probes), diluted 350-fold with TE buffer, was added to quantitate the double-stranded RNA produced during the reaction. Fluorescence was measured with an EnVision plate reader (PerkinElmer) at excitation and emission wavelengths of 485 nm and 510 nm, respectively. The RdRp activity for each well was calculated as follows: Percentage activity = 100 × (experimental read value − low control average value) / (high control average value − low control average value), where the high control value was obtained with 1% DMSO (without compounds) in the presence of 1 mM GTP, while the low control value was obtained with 1% DMSO (without compounds) in the absence of GTP. In this system, the signal-to-basal (S/B) ratio was 6.8, and the coefficient of variation (CV, high / low controls) values and Z’ factor were 5.4 / 5.3% and 0.78, respectively. In the first screening (n = 1), compounds that exhibited more than 40% inhibition (activities worse than 60% of the average high control) were selected. The selected compounds were then reexamined in triplicate under the same conditions, and the compounds with reproducibility were scored as hit compounds.

### Optimization of the reaction conditions

The MnCl_2_ concentrations, pH, temperature, protein concentrations, and reaction time were examined to optimize the RdRp assay conditions (S1A–S1D Fig). The reaction scale was 8 μl, and the reaction components are the same as those employed for the fragment screening, except that they did not include any compound and concentrations of RdRp or MnCl_2_ were varied as below. For all the conditions, experiments were performed in triplicate. The RdRp activities remained relatively constant with the examined MnCl_2_ concentrations (0.05–5 mM) (S1A Fig). As precipitations occurred in reaction mixtures with more than 10 mM MnCl_2_, 5 mM MnCl_2_ was employed for the RdRp assays. The DENV1-4 NS5 proteins, but not DENV2 RdRp, exhibited slightly higher RdRp activities at room temperature as compared with those at 37°C (S1A and S1B Fig). Analyses of the pH dependence revealed that the full-length NS5 proteins and DENV2 RdRp exhibited the highest activity at neutral pH, as compared with acidic and alkaline pH conditions (S1C Fig). Finally, the RdRp activities were dependent on the protein concentrations, and the reaction time courses (hours) are almost linear (S1D Fig). No significant difference was observed in the enzymatic activity among the DENV1-4 NS5 proteins. As precipitation appeared under conditions with > 5 μM protein, a 1 μM protein concentration was chosen for the RdRp assays.

### Determination of IC_50_

The RdRp assay for the IC_50_ determination was performed by using the full-length NS5 proteins (DENV1-4). Each reaction was performed in a 12 μl reaction. 3 μl of a 4 μM NS5 protein dissolved in the reaction buffer was mixed with 3 μl of various concentrations of the compound dissolved in 4% DMSO, and the mixture was incubated at room temperature for 60 min. Subsequently, 3 μl of 250 nM poly-C dissolved in water was added, and the mixture was incubated at room temperature for 60 min. The reactions were initiated by adding 3 μl of 4 mM GTP to the mixture. The final concentrations of the reaction components are 1 μM RdRp, 62.5 nM poly-C, 1mM GTP, 1% DMSO, 20 mM Tris-HCl (pH 7.4), 11.9 mM NaCl, 7.5% glycerol, 5 mM MnCl_2_, and 7 mM 2-mercaptoethanol, and various concentrations of compounds. After incubating at room temperature for 2.5 hours, 75 μl of PicoGreen Quantitation Reagent, diluted 116-fold with TE buffer, was added to quantitate the produced double-stranded RNA. For these experiments, a Zephyr Robotic system equipped with an ARVO plate reader (PerkinElmer) was employed. Fluorescence was measured at excitation and emission wavelengths of 485 nm and 535 nm, respectively. In this system, the signal-to-basal (S/B) ratio was 5.4, and the CV values (high / low controls) and Z’ factor were 8.8 / 8.4% and 0.61, respectively. The experiments were performed in quadruplicate. The IC_50_ values and p-values were determined by fitting a five-parameter logistic curve to the data, using PRISM version 6 (GraphPad Software, Inc.).

### Crystallization and X-ray crystallography of the NS5 RdRp domains

Crystallization of the NS5 RdRp domains was performed by the sitting-drop vapor diffusion method. For the crystallization of the DENV2 RdRp, 0.3 μl of the protein solution was mixed with 0.3 μl of reservoir solution, containing 1.5–1.6 M ammonium sulfate, 100 mM MES-NaOH (pH 6.2–6.5), and 10 mM CoCl_2_, and the mixture was equilibrated with the reservoir at 20 ºC. Crystallization of the DENV3 RdRp was achieved in a similar manner, using a reservoir solution containing 16% polyethylene glycol (PEG) 10,000 and 100 mM Tris-HCl (pH 8.0). Prior to data collection, the crystals were soaked at room temperature overnight in reservoir solutions containing a saturated concentration of RK-0404678, transferred to the reservoir solutions containing 28% glycerol as a cryoprotectant, and flash cooled at 100 K in a nitrogen-gas stream. X-ray diffraction data were collected at 100 K, using synchrotron radiation with a wavelength of 1.0 Å, at BL26B2 in SPring-8 [33]. All data were integrated and scaled using the Xia2 pipeline [34]. The structures were solved by molecular replacement with the program PHENIX [35], using the DENV3 RdRp structure (PDB ID: 2J7U) as the search model. Model building and refinement were performed with the programs COOT [36] and PHENIX [35]. Data collection and refinement statistics are listed in S1 Table.

### Cell culture

Vero cells were cultured in modified Eagle’s medium (MEM) (Nacalai Tesque), supplemented with 10% fetal bovine serum (FBS) (HyClone), 1x Non-Essential Amino Acids Solution (NEAA, Thermo), and 1x penicillin-streptomycin (P/S, Nacalai Tesque). BHK-21 cells were cultured in MEM (Nacalai Tesque), supplemented with 5% FBS, 1x NEAA, and 1x P/S. C6/36 cells were cultured at 28°C in Leibovitz L-15 medium (Thermo), supplemented with 10% FBS, 0.3% tryptose phosphate broth (TPB), and 1x P/S.

### Viruses

Dengue virus serotype 1 (DENV1) (Mochizuki strain), DENV2 (16681 strain and P04/08 strain), DENV3 (H87 strain), and DENV4 (H241 strain) were kind gifts from Dr. T. Kurosu. The DENV2 (P04/08 strain) was described previously [37]. These viruses were propagated in C6/36 cells. Viral titers were determined with an RT-qPCR assay.

### Cytotoxicity assay

The cytotoxic activities of the compounds were tested using a WST-1 assay (Roche Diagnostics), according to the manufacturer’s instructions. Briefly, Vero cells plated at 7.5 x 10^3^ cells per well of a 96-well plate were cultured with serially diluted compounds for 2 days. Ten μL of WST-1 solution was added to each well, and the cells were further incubated for 30 min. The absorbance was measured at 450 nm, with subtraction of the nonspecific background at 650 nm. Concentrations that showed comparable absorbance to the DMSO control were judged to be non-cytotoxic.

### Infection

Vero cells were plated at 1 x 10^4^ cells per well of a 96-well plate and incubated overnight prior to infection. The following day, the cells were infected with dengue viruses for 2 hours with an MOI of 0.5. The cells were vigorously washed and then cultured in MEM supplemented with 2% FBS, 1x NEAA, 1x P/S, and the compounds. The viral titers in the culture supernatants were determined at 3 days after infection, using an RT-qPCR assay. The degree of inhibition by the compounds was calculated by dividing the copy numbers of the culture supernatants in the presence of compounds by those in the absence of compounds (only DMSO).

### Quantification of viral RNA

Viral RNA was isolated from the culture supernatant using a QIAmp Viral RNA Mini kit (Qiagen) or a NucleoSpin 8 Virus kit (TaKaRa), according to the manufacturers’ protocols. Viral RNA was quantified using a One-Step SYBR PrimeScript RT-PCR kit II (TaKaRa) and the following universal primers for dengue viruses: DNF, 5´- CTWTCAATATGCTGAAACGCG-3´ and DNR, 5´- TCTATCCARAATYCCTGCTGTT-3´ [38]. The total reaction volume was 12.5 μL per tube. The PCR conditions were 42°C for 5 min and 95°C for 10 min for reverse transcription, followed by 45 cycles of 95°C for 5 s, 55°C for 30 s, and 72°C for 30 s. The fluorescent signals were detected with a 7500 Fast Real-Time PCR System (Applied Biosystems).

### Determination of EC_50_

The experiments were performed in triplicate. The EC_50_ values and p-values were determined by fitting a five-parameter logistic curve on the data, using PRISM version 6 (GraphPad Software, Inc.).

### Adaptation of DENV2 in RK-0404678-treated Vero cells

Vero cells, plated at 4.2 x 10^4^ cells per well of a 6-well plate, were incubated overnight prior to infection. For infection, the cells were infected with DENV2 16681 at an MOI of 0.1. The cells were vigorously washed and then cultured for 7 days in the presence of either 50 μM or 25 μM RK-0404678. For the adaptation of the virus to RK-0404678, 300 μL portions of virus-containing supernatants were used to infect fresh Vero target cells. The cells were cultured for 7 days in the presence of RK-0404678. These passages were repeated 9 times in total.

### Rescue of recombinant viruses

Recombinant viruses harboring mutations in the NS5 gene were generated with the CPER method, as reported previously [39]. Briefly, the whole genome of the DENV2 16681 strain was PCR amplified to generate six fragments, harboring overlapping sequences with adjacent fragments. PCR fragments were generated with high-fidelity PrimeSTAR GXL DNA polymerase (TaKaRa) and primer pairs that have complementary ends with a 25-nucleotide overlap (S2 Table). PCR fragments were cloned into the pCR2.1-TOPO vector (Thermo) and verified by sequencing. In addition, the DNA fragment encoding a polyA signal, the hepatitis delta virus ribozyme (HDVr), and the minimal CMV promoter was synthesized and cloned into the pCR-TOPO vector to generate the pCR-TOPO-pCMV-RZM plasmid (kind gift from Dr. T. Okamoto). Mutations in the NS5 gene were introduced with the standard overlapping PCR method. The seven DNA fragments were prepared by PCR, mixed in equimolar amounts (0.1 pmol each), and subjected to CPER with PrimeSTAR GXL DNA polymerase (TaKaRa) (an initial 2 min of denaturation at 98°C; 20 cycles of 10 s at 98°C, 15 s at 55°C, and 12 min at 68°C; and a final extension for 12 min at 68°C) to generate circular DNA. The CPER products were then used for transfection into BHK-21 cells with the TransIT-LT1 Reagent (TaKaRa), according to the manufacturer’s instructions. At 6 days after transfection, the culture supernatants were treated with TURBO DNase (Thermo) and the viral titers were determined by an RT-qPCR assay. The viruses were propagated in C6/36 cells for further experiments.

### Accession codes

The coordinates and structure factors have been deposited in the Protein Data Bank, under the accession codes 6IZX and 6IZY for the RK-0404678-bound and apo forms of the DENV2 RdRp, and 6IZZ and 6J00 for the RK-0404678-bound and apo forms of the DENV3 RdRp, respectively. Authors will release the atomic coordinates and experimental data upon article publication.

## Supporting information

S1 Fig Optimization of the RdRp assay conditions. A-B. Effects of the MnCl_2_ concentration on the double-stranded RNA synthesis were examined for the full-length NS5 and RdRp proteins. Reactions were performed for 120 min at room temperature (A) and at 37°C (B). The double-stranded RNA formation was measured using PicoGreen. C. The effects of double-stranded RNA synthesis using different buffers (MES (pH 6.0) / Tris-HCl (pH 7.4) / Tris-HCl (pH 8.8)) were examined. D. Enzyme concentration dependencies of the double-stranded RNA synthesis were examined and are shown as reaction time courses (hours). The results shown are the mean and standard deviation of triplicate measurements.
(PDF)

S2 Fig HTS results for the fragment library. A-B. The first screening of all fragment compounds (A), and re-examination of the compounds that showed > 40% inhibition (B). The vertical axis shows the RdRp activity (%) in the presence of each fragment compound (100 μM), normalized to the controls (1% DMSO ± GTP). The horizontal axis represents the identification number arbitrarily assigned to each fragment compound. RK-0404678 is indicated in red. The results of the first screening shown in (A) is from a single experiment. The results shown in (B) are the mean and standard deviation of triplicate measurements for each compound.
(PDF)

S3 Fig Longitudinal antiviral effect of RK-0404678. Vero cells were infected with either the DENV-2 16681 or P04/08 strain in the presence of RK-0404678. The viral RNA in the culture supernatant was measured at 24, 48, and 72 hours after infection (left). The sensitivity to RK-0404678 is displayed as the relative value normalized to control cells without the compound treatment at 72 hours after infection (right). The results shown are the mean and standard deviation of triplicate measurements.
(PDF)

S4 Fig Cys residues in contact with RK-0404678. A. Cys780 in DENV2 Site 1. B. Cys709 in DENV2 Site 2. C. Cys780 in DENV3 Site 1. D. Positions of the Cys780 and Cys709 residues in Sites 1 and 2 in DENV2. Their side chains are colored red, and the RK-0404678 molecules are magenta.
(PDF)

S5 Fig Conservation of the Cys709 and Cys780 residues. WebLogo representation of the sequence conservation of NS5 residues. The NS5 sequences of 219 independent DENV1-4 viruses were analyzed. The height of a particular residue indicates its degree of conservation. The Cys709 and Cys780 residues are highly conserved in DENV1-4.
(PDF)

S6 Fig NS5 mutant viruses were rescued by transfecting BHK-21 cells with CPER products. The viral titer in the culture supernatant was evaluated by RT-qPCR.
(PDF)

S7 Fig Protein sequence alignment of the full-length DENV1-4 NS5 proteins used in this study. The alignment was performed using the MultAlin program (http://multalin.Toulouse.inra.fr/multalin/multalin.html). The high-, low-, and neutral-consensus amino acid residues are depicted in red, blue, and black colors according to the MultAlin program, respectively. The DENV2 RdRp protein (a.a. 251–896) used for the crystallographic analyses and the fragment screening contains G321V and K891R substitutions (the same sequence as in PDB ID: 5K5M [11]).
(PDF)

S8 Fig Sensitivity of the RK-0404678-adapted (P9) virus to RK-0404678. The viral titer in the culture supernatant was evaluated by RT-qPCR. The results shown are the mean and standard deviation of triplicate measurements.
(PDF)

S9 Fig Synthesized DNA sequences of the full-length DENV1-4 NS5 proteins used in this study. (PDF)

S10 Fig SDS-PAGE analyses of the purified recombinant RdRp proteins and full-length NS5 proteins. The purification processes of the recombinant DENV2 and 3 RdRp proteins are shown in the upper panels. The eluted fractions of the full-length NS5 proteins from Superdex200 are shown in the lower panels. The gels were stained with Coomassie Brilliant Blue.
(PDF)

S1 Table Data collection and refinement statistics. (PDF)

S2 Table List of primers used in this study. (PDF)
