# Supplementary material for: Discovery of a small molecule inhibitor targeting dengue virus NS5 RNA-dependent RNA polymerase
Source: PLoS Negl Trop Dis. 2019 Nov 18;13(11):e0007894. doi: 10.1371/journal.pntd.0007894 (PMC6886872; doi:10.1371/journal.pntd.0007894)
Supplement: S1 Fig — A-B. Effects of the MnCl2 concentration on the double-stranded RNA synthesis were examined for the full-length NS5 and RdRp proteins. Reactions were performed for 120 min at room temperature (A) and at 37°C (B). The double-stranded RNA formation was measured using PicoGreen. C. The effects of double-stranded RNA synthesis using different buffers (MES (pH 6.0) / Tris-HCl (pH 7.4) / Tris-HCl (pH 8.8)) were examined. D. Enzyme concentration dependencies of the double-stranded RNA synthesis were examined and are shown as reaction time courses (hours). The results shown are the mean and standard deviation of triplicate measurements. (PDF) [file pntd.0007894.s001.pdf]

S1 Fig.

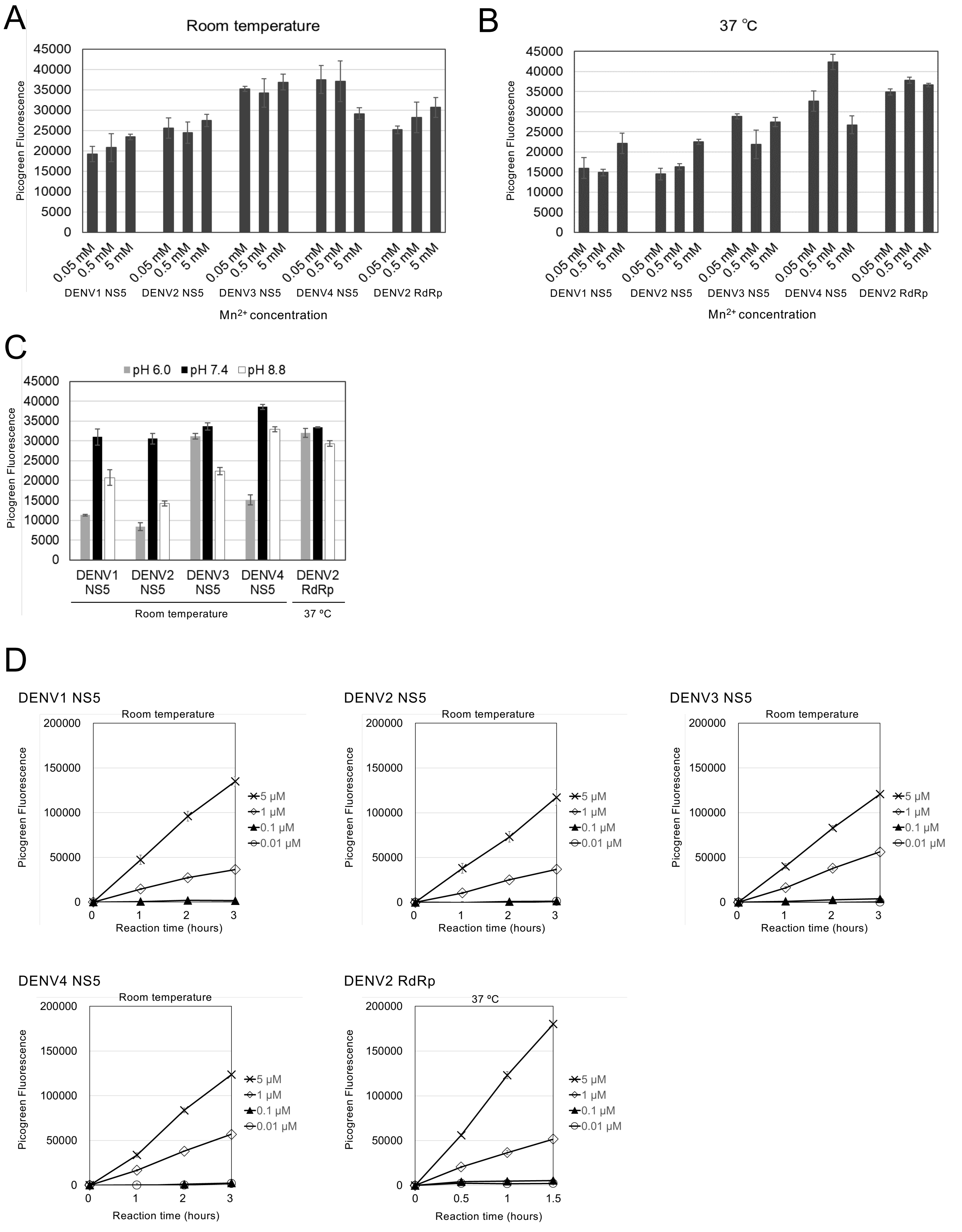
A
Room temperature
Picogreen Fluorescence
45000
40000
35000
30000
25000
20000
15000
10000
5000
0
0.05 mM
0.5 mM
5 mM
DENV1 NS5
DENV2 NS5
DENV3 NS5
DENV4 NS5
DENV2 RdRp
Mn2+ concentration
B
37 °C
Picogreen Fluorescence
0.05 mM
0.5 mM
5 mM
DENV1 NS5
DENV2 NS5
DENV3 NS5
DENV4 NS5
DENV2 RdRp
Mn2+ concentration
C
pH 6.0
pH 7.4
pH 8.8
Picogreen Fluorescence
DENV1 NS5
DENV2 NS5
DENV3 NS5
DENV4 NS5
DENV2 RdRp
Room temperature
37 °C
D
DENV1 NS5
DENV2 NS5
DENV3 NS5
DENV4 NS5
DENV2 RdRp
Room temperature
37 °C
Picogreen Fluorescence
200000
150000
100000
50000
0
5 μM
1 μM
0.1 μM
0.01 μM
Reaction time (hours)
0
0.5
1
1.5
2
3

S1 Fig. (continued)

S1 Fig. Optimization of the RdRp assay conditions. A-B. Effects of the $MnCl_2$ concentration on the double-stranded RNA synthesis were examined for the full-length NS5 and RdRp proteins. Reactions were performed for 120 min at room temperature (A) and at 37 ºC (B). The double-stranded RNA formation was measured using PicoGreen. C. The effects of double-stranded RNA synthesis using different buffers (MES (pH 6.0) / Tris-HCl (pH 7.4) / Tris-HCl (pH 8.8)) were examined. D. Enzyme concentration dependencies of the double-stranded RNA synthesis were examined and are shown as reaction time courses (hours). The results shown are the mean and standard deviation of triplicate measurements.
