# Supplementary material for: Discovery of a small molecule inhibitor targeting dengue virus NS5 RNA-dependent RNA polymerase
Source: PLoS Negl Trop Dis. 2019 Nov 18;13(11):e0007894. doi: 10.1371/journal.pntd.0007894 (PMC6886872; doi:10.1371/journal.pntd.0007894)
Supplement: S2 Fig — A-B. The first screening of all fragment compounds (A), and re-examination of the compounds that showed > 40% inhibition (B). The vertical axis shows the RdRp activity (%) in the presence of each fragment compound (100 μM), normalized to the controls (1% DMSO ± GTP). The horizontal axis represents the identification number arbitrarily assigned to each fragment compound. RK-0404678 is indicated in red. The results of the first screening shown in (A) is from a single experiment. The results shown in (B) are the mean and standard deviation of triplicate measurements for each compound. (PDF) [file pntd.0007894.s002.pdf]

S2 Fig.

A

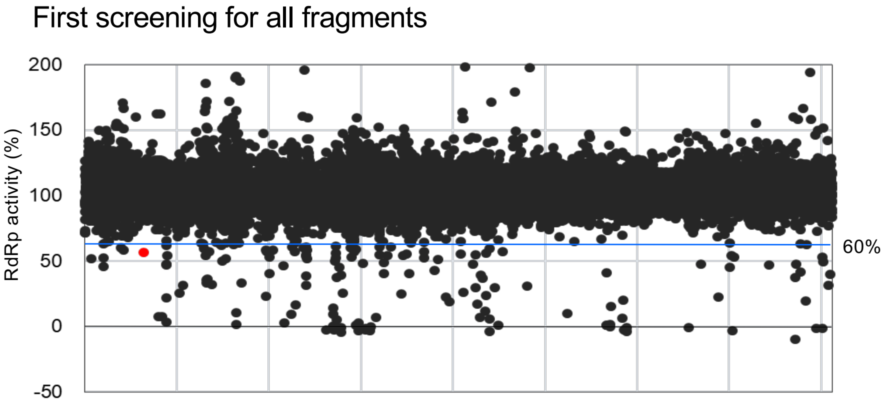

B

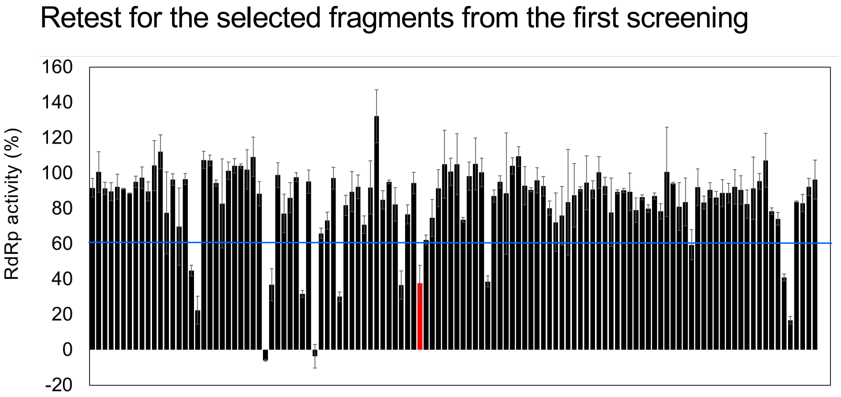

S2 Fig. HTS results for the fragment library. A-B. The first screening of all fragment compounds (A), and re-examination of the compounds that showed > 40% inhibition (B). The vertical axis shows the RdRp activity (%) in the presence of each fragment compound (100 μM), normalized to the controls (1% DMSO ± GTP). The horizontal axis represents the identification number arbitrarily assigned to each fragment compound. RK-0404678 is indicated in red. The results of the first screening shown in (A) is from a single experiment. The results shown in (B) are the mean and standard deviation of triplicate measurements for each compound.
