# Supplementary material for: Discovery of a small molecule inhibitor targeting dengue virus NS5 RNA-dependent RNA polymerase
Source: PLoS Negl Trop Dis. 2019 Nov 18;13(11):e0007894. doi: 10.1371/journal.pntd.0007894 (PMC6886872; doi:10.1371/journal.pntd.0007894)
Supplement: S3 Fig — Vero cells were infected with either the DENV-2 16681 or P04/08 strain in the presence of RK-0404678. The viral RNA in the culture supernatant was measured at 24, 48, and 72 hours after infection (left). The sensitivity to RK-0404678 is displayed as the relative value normalized to control cells without the compound treatment at 72 hours after infection (right). The results shown are the mean and standard deviation of triplicate measurements. (PDF) [file pntd.0007894.s003.pdf]

S3 Fig.

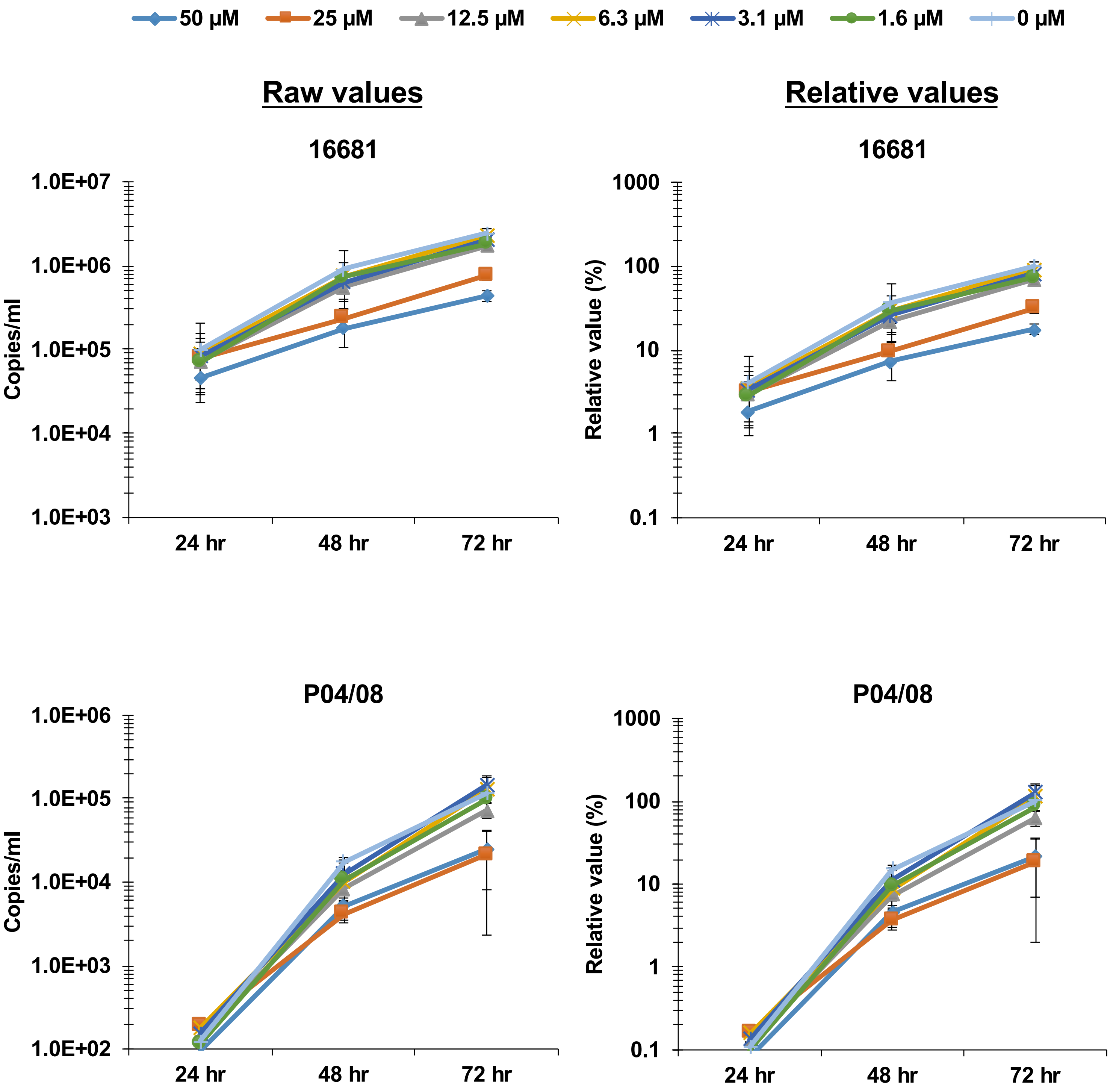

S3 Fig. Longitudinal antiviral effect of RK-0404678. Vero cells were infected with either the DENV-2 16681 or P04/08 strain in the presence of RK-0404678. The viral RNA in the culture supernatant was measured at 24, 48, and 72 hours after infection (left). The sensitivity to RK-0404678 is displayed as the relative value normalized to control cells without the compound treatment at 72 hours after infection (right). The results shown are the mean and standard deviation of triplicate measurements.
