# Supplementary material for: Discovery of a small molecule inhibitor targeting dengue virus NS5 RNA-dependent RNA polymerase
Source: PLoS Negl Trop Dis. 2019 Nov 18;13(11):e0007894. doi: 10.1371/journal.pntd.0007894 (PMC6886872; doi:10.1371/journal.pntd.0007894)
Supplement: S4 Fig — A. Cys780 in DENV2 Site 1. B. Cys709 in DENV2 Site 2. C. Cys780 in DENV3 Site 1. D. Positions of the Cys780 and Cys709 residues in Sites 1 and 2 in DENV2. Their side chains are colored red, and the RK-0404678 molecules are magenta. (PDF) [file pntd.0007894.s004.pdf]

S4 Fig.

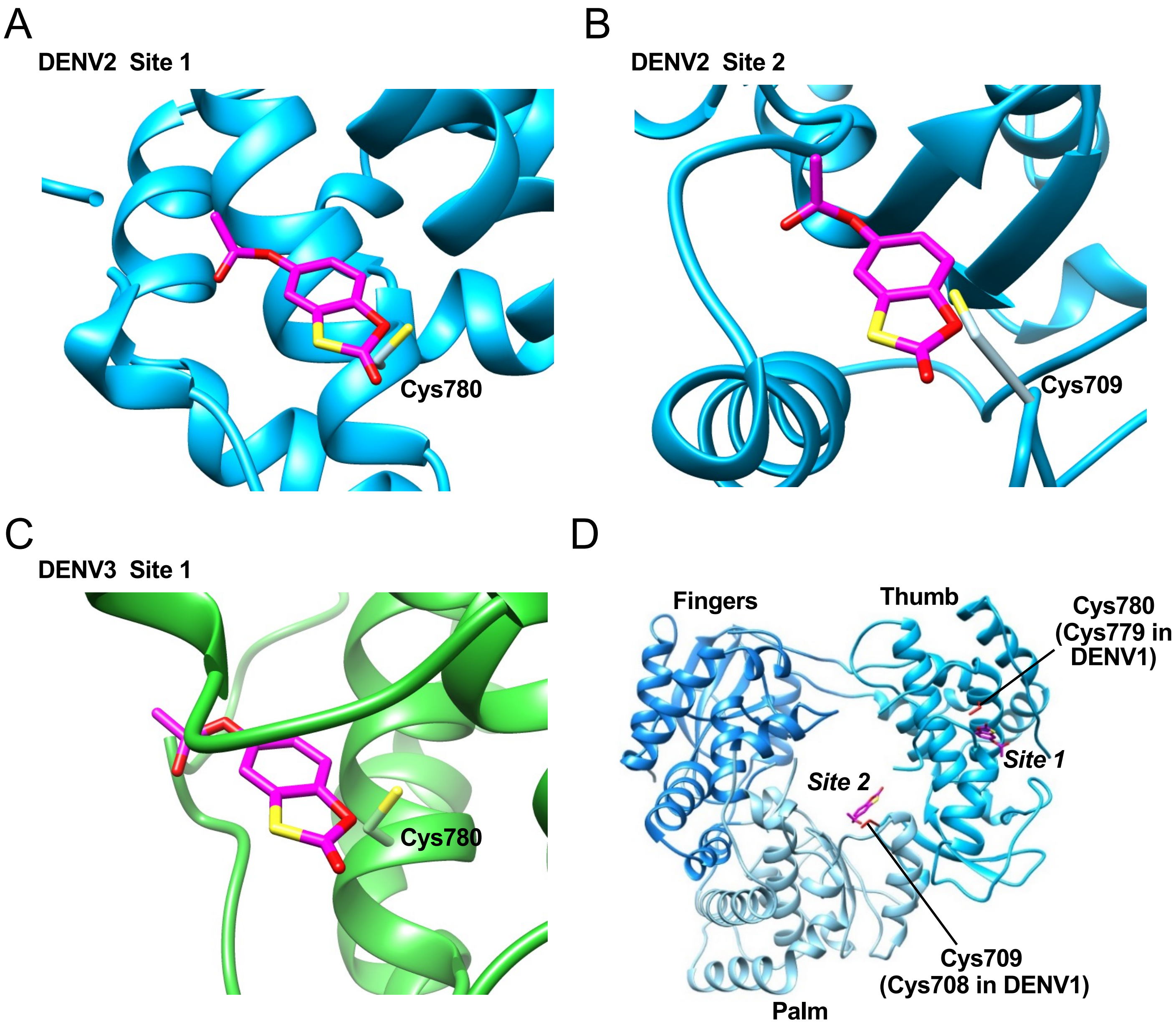

S4 Fig. Cys residues in contact with RK-0404678. A. Cys780 in DENV2 Site 1. B. Cys709 in DENV2 Site 2. C. Cys780 in DENV3 Site 1. D. Positions of the Cys780 and Cys709 residues in Sites 1 and 2 in DENV2. Their side chains are colored red, and the RK-0404678 molecules are magenta.
