# Supplementary material for: Discovery of a small molecule inhibitor targeting dengue virus NS5 RNA-dependent RNA polymerase
Source: PLoS Negl Trop Dis. 2019 Nov 18;13(11):e0007894. doi: 10.1371/journal.pntd.0007894 (PMC6886872; doi:10.1371/journal.pntd.0007894)
Supplement: S5 Fig — WebLogo representation of the sequence conservation of NS5 residues. The NS5 sequences of 219 independent DENV1-4 viruses were analyzed. The height of a particular residue indicates its degree of conservation. The Cys709 and Cys780 residues are highly conserved in DENV1-4. (PDF) [file pntd.0007894.s005.pdf]

S5 Fig.

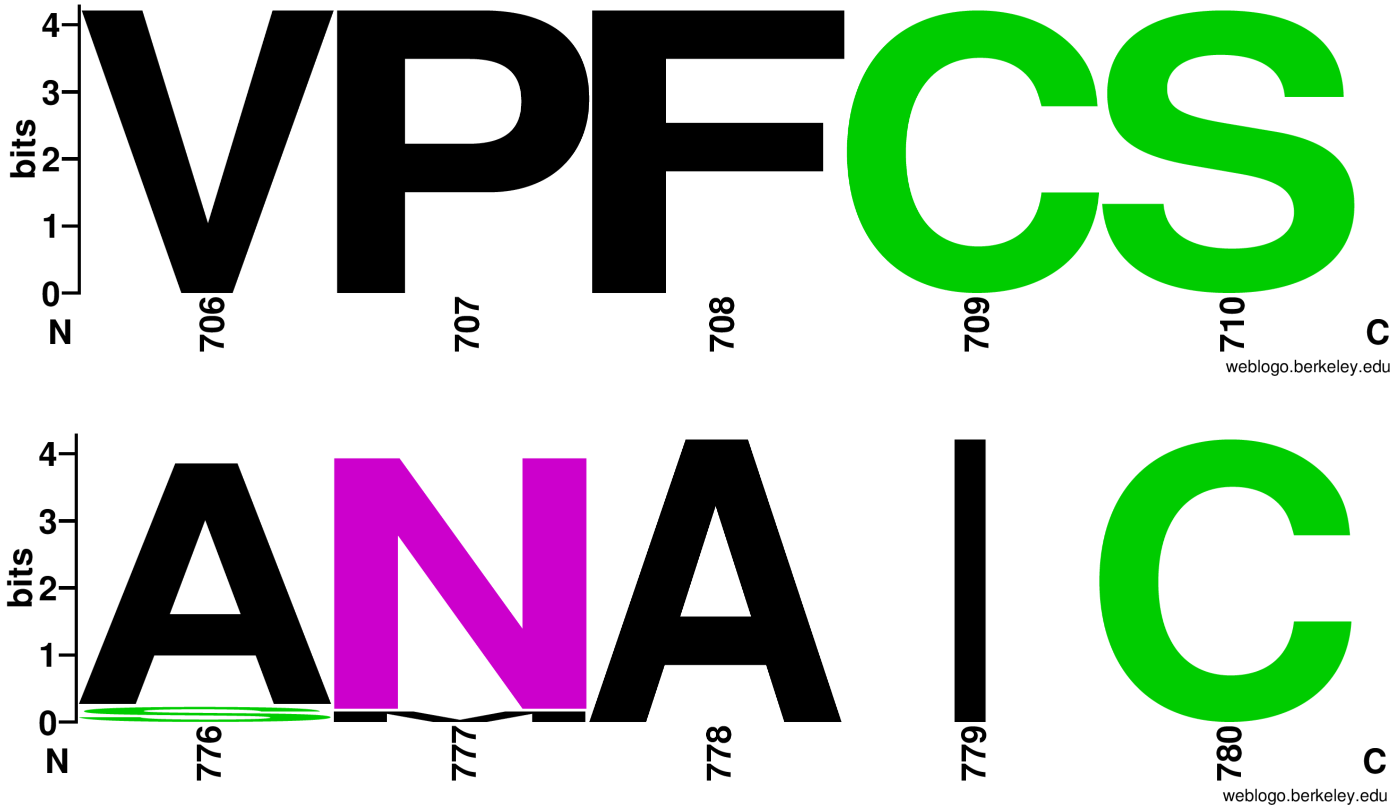

S5 Fig. Conservation of the Cys709 and Cys780 residues. WebLogo representation of the sequence conservation of NS5 residues. The NS5 sequences of 219 independent DENV1-4 viruses were analyzed. The height of a particular residue indicates its degree of conservation. The Cys709 and Cys780 residues are highly conserved in DENV1-4.
