# Supplementary material for: Discovery of a small molecule inhibitor targeting dengue virus NS5 RNA-dependent RNA polymerase
Source: PLoS Negl Trop Dis. 2019 Nov 18;13(11):e0007894. doi: 10.1371/journal.pntd.0007894 (PMC6886872; doi:10.1371/journal.pntd.0007894)
Supplement: S6 Fig — The viral titer in the culture supernatant was evaluated by RT-qPCR. (PDF) [file pntd.0007894.s006.pdf]

S6 Fig.

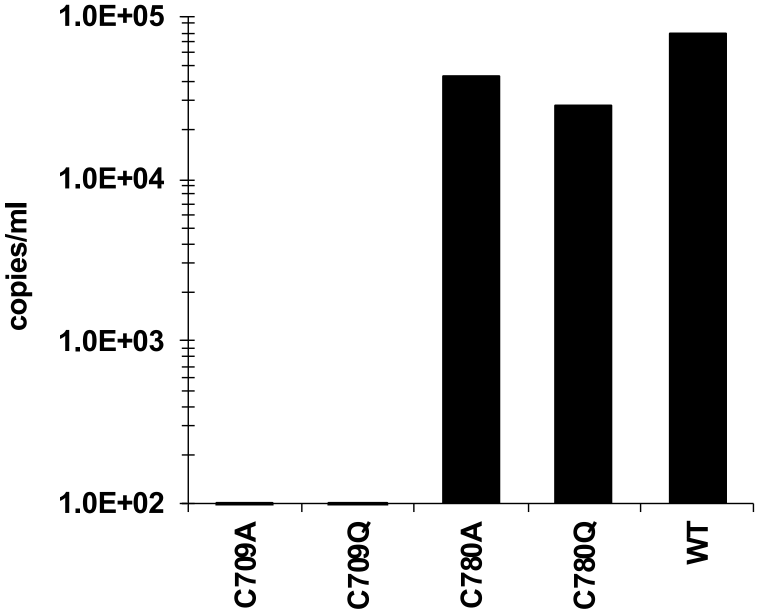

S6 Fig. NS5 mutant viruses were rescued by transfecting BHK-21 cells with CPER products. The viral titer in the culture supernatant was evaluated by RT-qPCR.
