# Supplementary material for: Discovery of a small molecule inhibitor targeting dengue virus NS5 RNA-dependent RNA polymerase
Source: PLoS Negl Trop Dis. 2019 Nov 18;13(11):e0007894. doi: 10.1371/journal.pntd.0007894 (PMC6886872; doi:10.1371/journal.pntd.0007894)
Supplement: S7 Fig — The alignment was performed using the MultAlin program (http://multalin.Toulouse.inra.fr/multalin/multalin.html). The high-, low-, and neutral-consensus amino acid residues are depicted in red, blue, and black colors according to the MultAlin program, respectively. The DENV2 RdRp protein (a.a. 251–896) used for the crystallographic analyses and the fragment screening contains G321V and K891R substitutions (the same sequence as in PDB ID: 5K5M [11]). (PDF) [file pntd.0007894.s007.pdf]

S7 Fig.

```
DENV1 NS5   1 ---------- -GTGAQGETL GEKWKRQLNQ LSKSEFNTYK RSGIIEVDRS EAKEGLKRGE PTKHAVSRGT AKLRWFVERN
DENV2 NS5   1 ---------- -GTGNIGETL GEKWKSRLNA LGKSEFQIYK KSGIQEVDRT LAKEGIKRGE TDHHAVSRGS AKLRWFVERN
DENV3 NS5   1 ---------- -GTGSQGETL GEKWKKRLNQ LSRKEFDLYK KSGITEVDRT EAKEGLKRGE ITHHAVSRGS AKLQWFVERN
DENV4 NS5 -11 SLIKNAQTPR RGTGTTGETL GEKWKRQLNS LDRKEFEEYK RSGILEVDRT EAKSALKDGS KIKYAVSRGT SKIRWIVERG

DENV1 NS5     LVKPEGKVID LGCGRGGWSY YCAGLKKVTE VKGYTKGGPG HEEPIPMATY GWNLVKLYSG KDVFFTPPEK CDTLLCDIGE
DENV2 NS5     MVTPEGKVVD LGCGRGGWSY YCGGLKNVRE VKGLTKGGPG HEEPIPMSTY GWNLVRLQSG VDVFFTPPEK CDTLLCDIGE
DENV3 NS5     MVIPEGRVID LGCGRGGWSY YCAGLKKVTE VRGYTKGGPG HEEPVPMSTY GWNIVKLMSG KDVFYLPPEK CDTLLCDIGE
DENV4 NS5     MVKPKGKVVD LGCGRGGWSY YMATLKNVTE VKGYTKGGPG HEEPIPMATY GWNLVKLHSG VDVFYKPTEQ VDTLLCDIGE

DENV1 NS5     SSPNPTIEEG RTLRVLKMVE PWLRGN-QFC IKILNPYMPS VVETLEQMQR KHGGMLVRNP LSRNSTHEMY WVSCGTGNIV
DENV2 NS5     SSPNPTVEAG RTLRVLNLVE NWLNNNTQFC IKVLNPYMPS VIEKMEALQR KYGGALVRNP LSRNSTHEMY WLSNASGNIV
DENV3 NS5     SSPSPTVEES RTIRVLKMVE PWLKNN-QFC IKVLNPYMPT VIEHLERLQR KHGGMLVRNP LSRNSTHEMY WISNGTGNIV
DENV4 NS5     SSSNPTIEEG RTLRVLKMVE PWLSSKPEFC IKVLNPYMPT VIEELEKLQR KHGGSLVRCP LSRNSTHEMY WVSGVSGNIV

DENV1 NS5     SAVNMTSRML LNRFTMAHRK PTYERDVDLG AGTRHVAVEP EVANLDIIGQ RIENIKNGHK STWHYDEDNP YKTWAYHGSY
DENV2 NS5     SSVNMISRML INRFTMRHKK ATYEPDVDLG SGTRNIGIES EIPNLDIIGK RIEKIKQEHE TSWHYDQDHP YKTWAYHGSY
DENV3 NS5     SSVNMVSRLL LNRFTMTYRR PTIEKDVDLG AGTRHVNAEP ETPNMDVIGE RIRRIKEEHS STWHYDDENP YKTWAYHGSY
DENV4 NS5     SSVNTTSKML LNRFTTRHRK PTYEKDADLG AGTRSVSTET EKPDMTIIGR RLQRLQEEHK ETWHYDHENP YRTWAYHGSY

DENV1 NS5     EVKPSGSASS MVNGVVRLLT KPWDVIPMVT QIAMTDTTPF GQQRVFKEKV DTRTPKAKRG TAQIMEVTAR WLWGFLSRNK
DENV2 NS5     ETKQTGSASS MGNGVVRLLT KPWDVVPMVT QMAMTDTTPF GQQRVFKEKV DTRTQEPKEG TKKLMKITAE WLWKELGKKK
DENV3 NS5     EVKATGSASS MINGVVKLLT KPWDVVPTVT QMAMTDTTPF GQQRVFKEKV DTRTPKPMPG TRKVMEITAG WLWRTLGRNK
DENV4 NS5     EAPSTGSASS MVNGVVKLLT KPWDVVPMVT QLAMTDTTPF GQQRVFKEKV DTRTPQPKPG TRVVMTTTAN WLWALLGRKK

DENV1 NS5     KPRICTREEF TRKVRSNAAI GAVFVDENQW NSAKEAVEDE RFWDLVHRER ELHKQGKCAT CVYNMMGKRE KKLGEFGKAK
DENV2 NS5     TPRMCTREEF TRKVRSNAAL GAIFTDENKW KSAREAVEDS RFWELVDKER NLHLEGKCET CVYNMMGKRE KKLGEFGKAK
DENV3 NS5     RPRLCTREEF TKKVRTNAAM GAVFTEENQW DSARAAVEDE EFWKLVDRER ELHKQGKCGS CVYNMMGKRE KKLGEFGKAK
DENV4 NS5     NPRLCTREEF ISKVRSNAAI GAVFQEEQGW TSASEAVNDS RFWELVDKER ALHQEGKCES CVYNMMGKRE KKLGEFGRAK

DENV1 NS5     GSRAIWYMWL GARFLEFEAL GFMNEDHWFS RENSLSGVEG EGLHKLGYIL RDISKIPGGN MYADDTAGWD TRITEDDLQN
DENV2 NS5     GSRAIWYMWL GARFLEFEAL GFLNEDHWFS RENSLSGVEG EGLHKLGYIL RDVSKKEGGA MYADDTAGWD TRITLEDLKN
DENV3 NS5     GSRAIWYMWL GARYLEFEAL GFLNEDHWFS RENSYSGVEG EGLHKLGYIL RDISKIPGGA MYADDTAGWD TRITEDDLHN
DENV4 NS5     GSRAIWYMWL GARFLEFEAL GFLNEDHWFG RENSWSGVEG EGLHRLGYIL EDIDKKDGDL IYADDTAGWD TRITEDDLLN

DENV1 NS5     EAKITDIMEP EHALLATSIF KLTYQNKVVR VQRPAKNGTV MDVISRRDQR GSGQVGTYGL NTFTNMEAQL IRQMESEGIF
DENV2 NS5     EEMVTNHMEG EHKKLAEAIF KLTYQNKVVR VQRPTPRGTV MDIISRRDQR GSGQVGTYGL NTFTNMEAQL IRQMEGEGVF
DENV3 NS5     EEKITQQMDP EHRQLANAIF KLTYQNKVVK VQRPTPKGTV MDIISRKDQR GSGQVGTYGL NTFTNMEAQL IRQMEGEGVL
DENV4 NS5     EELITEQMAP HHKILAKAIF KLTYQNKVVK VLRPTPKGAV MDIISRKDQR GSGQVGTYGL NTFTNMEVQL IRQMEAEGVI

DENV1 NS5     SPSELETPNL AER-VLDWLK KHGTERLKRM AISGDDCVVK PIDDRFATAL TALNDMGKVR KDIPQWEPSK GWNDWQQVPF
DENV2 NS5     KSIQHLTVT- EEIAVQNWLA RVGRERLSRM AISGDDCVVK PLDDRFASAL TALNDMGKVR KDIQQWEPSR GWNDWTQVPF
DENV3 NS5     SKTDLENPHL LEKKITQWLE TKGVERLKRM AISGDDCVVK PIDDRFANAL LALNDMGKVR KDIPQWQPSK GWHDWQQVPF
DENV4 NS5     TRDDMHNPKG LKERVEKWLK ECGVDRLKRM AISGDDCVVK PLDERFSTSL LFLNDMGKVR KDIPQWEPSK GWKNWQEVPF

DENV1 NS5     CSHHFHQLIM KDGREIVVPC RNQDELVGRA RVSQGAGWSL RETACLGKSY AQMWQLMYFH RRDLRLAANA ICSAVPVDWV
DENV2 NS5     CSHHFHELIM KDGRVLVVPC RNQDELIGRA RISQGAGWSL RETACLGKSY AQMWSLMYFH RRDLRLAANA ICSAVPSHWV
DENV3 NS5     CSHHFHELIM KDGRKLVVPC RPQDELIGRA RISQGAGWSL KETACLGKAY AQMWALMYFH RRDLRLASNA ICSAVPVHWV
DENV4 NS5     CSHHFHKIFM KDGRSLVVPC RNQDELIGRA RISQGAGWSL RETACLGKAY AQMWSLMYFH RRDLRLASMA ICSAVPTEWF

DENV1 NS5     PTSRTTWSIH AHHQWMTTED MLSVWNRVWI EENPWMEDKT HVSSWEDVPY LGKREDRWCG SLIGLTARAT WATNIQVAIN
DENV2 NS5     PTSRTTWSIH AKHEWMTTED MLTVWNRVWI QENPWMEDKT PVESWEEIPY LGKREDQWCG SLIGLTSRAT WAKNIQTAIN
DENV3 NS5     PTSRTTWSIH AHHQWMTTED MLTVWNRVWI EDNPWMEDKT PVTTWEDVPY LGKREDQWCG SLIGLTSRAT WAQNILTAIQ
DENV4 NS5     PTSRTTWSIH AHHQWMTTED MLKVWNRVWI EDNPNMIDKT PVHSWEDIPY LGKREDLWCG SLIGLSSRAT WAKNIQTAIT

DENV1 NS5     QVRRLIGNEN YLDFMTSMKR FKNESDPEGA LW 899
DENV2 NS5     QVRSLIGNEE YTDYMPSMKR FRKEEEEAGV LW 900
DENV3 NS5     QVRSLIGNEE FLDYMPSMKR FRKEEESEGA IW 900
DENV4 NS5     QVRNLIGKEE YVDYMPVMKR YSAHFESEGV L- 900
```

S7 Fig. Protein sequence alignment of the full-length DENV1-4 NS5 proteins used in this study. The alignment was performed using the MultAlin program (http://multalin.Toulouse.inra.fr/multalin/multalin.html). The high-, low-, and neutral-consensus amino acid residues are depicted in red, blue, and black colors according to the MultAlin program, respectively. The DENV2 RdRp protein (a.a. 251-896) used for the crystallographic analyses and the fragment screening contains G321V and K891R substitutions (the same sequence as in PDB ID: 5K5M [11]).
