# Supplementary material for: Discovery of a small molecule inhibitor targeting dengue virus NS5 RNA-dependent RNA polymerase
Source: PLoS Negl Trop Dis. 2019 Nov 18;13(11):e0007894. doi: 10.1371/journal.pntd.0007894 (PMC6886872; doi:10.1371/journal.pntd.0007894)
Supplement: S8 Fig — The viral titer in the culture supernatant was evaluated by RT-qPCR. The results shown are the mean and standard deviation of triplicate measurements. (PDF) [file pntd.0007894.s008.pdf]

S8 Fig.

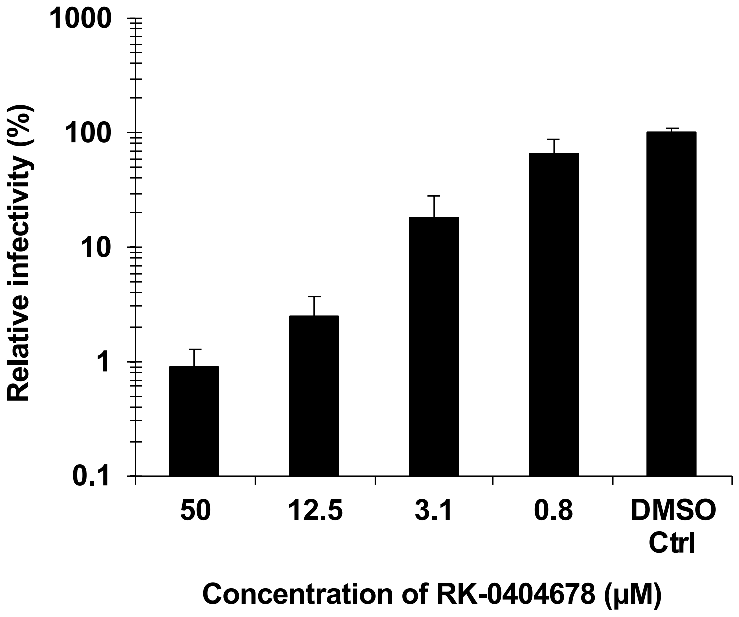

S8 Fig. Sensitivity of the RK-0404678-adapted (P9) virus to RK-0404678. The viral titer in the culture supernatant was evaluated by RT-qPCR. The results shown are the mean and standard deviation of triplicate measurements.
