# Supplementary material for: Discovery of a small molecule inhibitor targeting dengue virus NS5 RNA-dependent RNA polymerase
Source: PLoS Negl Trop Dis. 2019 Nov 18;13(11):e0007894. doi: 10.1371/journal.pntd.0007894 (PMC6886872; doi:10.1371/journal.pntd.0007894)
Supplement: S9 Fig — (PDF) [file pntd.0007894.s009.pdf]

S9 Fig.

DENV1

GGTACCGGTGCCCAAGGGGAAACCTTAGGTGAAAAATGGAAACGCCAGCTGAATCAGCTTTCCAAAAGCGAGTTCAACACGTATAAACGGTC
CGGAATTATCGAGGTCGATCGCTCTGAAGCAAAAGAAGGCTTGAAACGTGGTGAGCCGACCAAACATGCGGTAAGTCGCGGCACAGCGAAAT
TACGCTGGTTCGTGGAACGCAATCTGGTTAAACCAGAGGGTAAGGTTATCGATCTGGGTTGCGGCCGTGGTGGGTGGTCTTACTATTGTGCC
GGTCTGAAAAAAGTGACCGAAGTAAAAGGCTACACGAAAGGCGGTCCTGGTCACGAAGAACCGATTCCGATGGCCACGTACGGCTGGAACCT
GGTCAAACTGTATTCGGGCAAAGATGTCTTCTTTACGCCGCCGGAAAAATGCGATACGCTGCTGTGTGACATTGGAGAGTCTAGCCCAAACC
CGACCATTGAAGAAGGTCGTACGTTGCGCGTCCTGAAGATGGTGGAACCTTGGCTTCGTGGCAATCAGTTTTGCATCAAGATTTTAAACCCC
TACATGCCAAGCGTCGTGGAAACTCTTGAGCAAATGCAGCGCAAACATGGTGGCATGCTCGTTCGCAATCCGCTGTCTCGCAACTCAACCCA
CGAAATGTACTGGGTTAGCTGTGGTACCGGAAACATCGTATCGGCGGTGAATATGACTTCCCGTATGTTGCTGAATCGCTTTACGATGGCTC
ATCGTAAGCCGACGTATGAACGCGATGTCGATCTCGGCGCAGGGACCAGGCATGTGGCAGTCGAACCGGAAGTGGCCAATTTAGACATTATT
GGCCAGCGCATAGAAAACATCAAAAATGGCCATAAGAGTACATGGCACTATGATGAGGATAACCCCTATAAGACGTGGGCTTATCACGGTTC
GTATGAAGTGAAACCGAGTGGTTCAGCCTCGTCAATGGTAAATGGGGTGGTTCGCTTGTTAACCAAGCCTTGGGACGTGATCCCTATGGTTA
CCCAGATTGCTATGACTGATACGACCCCCTTTGGACAACAGCGCGTTTTCAAGGAGAAAGTCGATACACGGACCCCAAAAGCAAAACGCGGC
ACCGCCCAAATTATGGAAGTTACCGCTCGTTGGCTTTGGGGCTTTCTCTCGCGCAACAAAAAACCGCGTATATGCACACGTGAAGAATTTAC
GAGAAAAGTGCGGTCGAACGCCGCAATCGGTGCCGTGTTCGTGGACGAGAACCAGTGGAACAGCGCGAAAGAGGCTGTCGAAGACGAGCGCT
TTTGGGATTTGGTTCATCGTGAACGGGAACTGCACAAACAGGGCAAATGTGCCACTTGTGTGTACAACATGATGGGGAAACGCGAAAAAAAA
CTGGGCGAGTTTGGCAAAGCCAAGGGTAGCAGAGCGATTTGGTACATGTGGCTGGGCGCGCGATTTCTGGAGTTCGAAGCTCTGGGGTTTAT
GAATGAGGATCACTGGTTTAGCCGTGAAAACTCACTAAGCGGTGTTGAAGGGGAAGGTCTACATAAACTGGGCTATATCCTGCGTGACATTT
CCAAGATTCCGGGTGGCAACATGTACGCCGATGACACCGCAGGCTGGGATACCCGTATTACCGAGGACGACTTGCAGAATGAGGCTAAGATC
ACGGACATAATGGAACCGGAACATGCATTGCTTGCCACTAGTATTTTCAAACTCACGTACCAGAATAAAGTAGTCCGGGTTCAGCGACCGGC
GAAAAACGGGACTGTAATGGATGTCATTAGTCGTCGTGACCAACGTGGATCTGGACAAGTAGGGACCTATGGGCTGAATACCTTCACCAATA
TGGAAGCGCAGCTGATTAGACAAATGGAATCGGAAGGCATCTTTTCGCCAAGCGAACTGGAGACCCCAAACTTGGCTGAACGCGTCTTAGAC
TGGCTGAAAAAACATGGCACCGAACGTCTTAAGCGTATGGCAATCTCCGGTGATGATTGCGTGGTCAAGCCGATCGACGATCGTTTTGCGAC
AGCGCTGACTGCGCTGAACGATATGGGGAAAGTTCGCAAAGATATCCCGCAATGGGAACCAAGCAAAGGATGGAACGATTGGCAGCAGGTTC
CGTTTTGCAGTCACCATTTCCACCAGCTGATCATGAAAGATGGCCGTGAAATCGTGGTTCCGTGCCGGAATCAAGACGAGTTAGTAGGCCGC
GCCCGAGTTTCTCAAGGCGCTGGTTGGTCACTGCGTGAAACGGCATGTCTGGGTAAATCCTATGCGCAAATGTGGCAGCTCATGTACTTTCA
TCGTCGCGATTTACGTCTGGCAGCGAACGCCATTTGCAGTGCGGTGCCCGTGGATTGGGTTCCTACGTCACGCACAACCTGGAGCATTCACG
CGCATCATCAGTGGATGACCACGGAAGACATGCTGAGCGTGTGGAACCGCGTGTGGATCGAAGAAAATCCGTGGATGGAGGATAAAACACAC
GTGAGCTCTTGGGAAGATGTACCTTATCTTGGCAAACGAGAAGATCGTTGGTGTGGTTCCCTGATTGGCCTCACTGCACGTGCGACCTGGGC
GACTAATATCCAGGTGGCGATTAATCAGGTGCGCCGCCTGATTGGAAATGAGAACTATCTCGACTTCATGACTAGCATGAAACGCTTCAAGA
ACGAGAGTGATCCCGAAGGCGCGCTGTGG

DENV2

GGCACCGGCAACATTGGCGAAACCCTGGGCGAAAAATGGAAAAGCCGTCTGAACGCGCTGGGCAAAAGCGAATTTCAGATTTATAAGAAAAG
CGGCATTCAGGAAGTGGATCGTACCCTGGCGAAAGAAGGCATTAAACGTGGCGAAACCGATCATCATGCCGTGAGCCGTGGCAGCGCGAAAC
TGCGTTGGTTTGTGGAACGTAACATGGTGACCCCGGAAGGCAAAGTGGTGGATCTGGGCTGCGGCCGTGGCGGCTGGAGCTATTATTGCGGC
GGCCTGAAAAACGTGCGTGAAGTGAAAGGCCTGACCAAAGGCGGCCCGGGCCATGAAGAACCGATTCCGATGAGCACCTATGGCTGGAACCT
GGTGCGTCTGCAAAGCGGCGTGGATGTGTTTTTCACCCCGCCGGAAAAATGCGATACCCTGCTGTGCGATATTGGCGAAAGCAGCCCGAACC
CGACCGTGGAAGCGGGCCGTACCCTGCGTGTGCTGAACCTGGTGGAAAACTGGCTGAACAACAACACCCAGTTTTGCATTAAAGTGCTGAAC
CCGTATATGCCGAGCGTGATTGAGAAAATGGAAGCGCTGCAACGTAAATATGGCGGCGCGCTGGTGCGTAACCCGCTGAGCCGTAACAGCAC
CCATGAAATGTATTGGCTGAGCAACGCGAGCGGCAACATTGTGAGCAGCGTGAACATGATTAGCCGTATGCTGATTAACCGTTTTACCATGC
GTCATAAGAAAGCGACCTATGAACCGGATGTGGATCTGGGCAGCGGCACCCGTAACATTGGCATTGAAAGCGAAATTCCGAACCTGGATATT
ATTGGCAAACGTATTGAGAAAATTAAACAGGAACATGAAACCAGCTGGCATTATGATCAGGATCATCCGTATAAAACCTGGGCGTATCATGG
CAGCTATGAAACCAAACAGACCGGCAGCGCGAGCAGCATGGGCAACGGCGTGGTGCGTCTGCTGACCAAACCGTGGGATGTGGTGCCGATGG
TGACCCAGATGGCGATGACCGATACCACCCCGTTTGGCCAGCAGCGTGTGTTTAAAGAAAAAGTGGATACCCGTACCCAGGAACCGAAAGAA
GGCACCAAGAAACTGATGAAAATTACCGCGGAATGGCTGTGGAAAGAACTGGGCAAAAAGAAAACCCCGCGTATGTGCACCCGTGAAGAATT
TACCCGTAAAGTGCGTAGCAACGCGGCGCTGGGCGCGATTTTTACCGATGAAAACAAATGGAAAAGCGCGCGTGAAGCGGTGGAAGATAGCC
GTTTTTGGGAACTGGTGGATAAAGAACGTAACCTGCATCTGGAAGGCAAATGCGAAACCTGCGTGTATAACATGATGGGCAAACGTGAAAAG
AAACTGGGCGAATTTGGCAAAGCGAAAGGCAGCCGTGCGATTTGGTATATGTGGCTGGGCGCGCGTTTTCTGGAATTTGAAGCGCTGGGCTT
TCTGAACGAAGATCATTGGTTTAGCCGTGAAAACAGCCTGAGCGGCGTGGAAGGCGAAGGCCTGCATAAACTGGGCTATATTCTGCGTGATG
TGAGCAAGAAAGAAGGCGGCGCGATGTATGCGGATGATACCGCGGGCTGGGATACCCGTATTACCCTGGAAGACCTGAAAAACGAAGAAATG
GTGACCAACCACATGGAAGGCGAACATAAGAAACTGGCGGAAGCGATTTTTAAACTGACCTATCAGAATAAAGTGGTGCGTGTGCAGCGTCC
GACCCCGCGTGGCACCGTGATGGATATTATTAGCCGTCGTGATCAGCGTGGCAGCGGCCAAGTGGGCACCTATGGCCTGAACACCTTTACCA
ACATGGAAGCGCAGCTGATTCGTCAGATGGAAGGCGAAGGCGTGTTTAAAAGCATTCAGCATCTGACCGTGACCGAAGAAATTGCGGTGCAG
AACTGGCTGGCGCGTGTGGGCCGTGAACGTCTGAGCCGTATGGCGATTAGCGGCGATGATTGCGTGGTGAAACCGCTGGATGATCGTTTTGC
GAGCGCGCTGACCGCGCTGAACGATATGGGCAAAGTGCGTAAAGATATTCAGCAGTGGGAACCGTCTCGTGGCTGGAACGATTGGACCCAGG
TGCCGTTTTGCAGCCATCATTTTCATGAACTGATTATGAAAGATGGCCGTGTTCTGGTGGTGCCGTGCCGTAACCAGGATGAACTGATTGGT
CGTGCACGTATTAGCCAGGGCGCGGGTTGGAGCCTGCGTGAAACCGCATGCCTGGGTAAAAGCTATGCGCAGATGTGGAGCCTGATGTATTT
TCATCGTCGTGATCTGCGTCTGGCAGCAAACGCGATTTGCAGCGCGGTGCCGAGCCATTGGGTGCCGACCAGCCGTACCACCTGGAGCATTC
ATGCGAAACATGAATGGATGACCACCGAAGATATGCTGACCGTGTGGAACCGTGTGTGGATTCAGGAAAACCCGTGGATGGAAGATAAAACC
CCGGTGGAAAGCTGGGAAGAAATTCCGTATCTGGGCAAACGTGAAGATCAGTGGTGCGGCAGCCTGATTGGCCTGACCAGCCGTGCGACCTG
GGCGAAAAACATTCAGACCGCGATTAACCAAGTGCGTAGCCTGATTGGCAATGAAGAATATACCGATTATATGCCGAGCATGAAACGTTTTC
GTAAAGAAGAAGAAGAAGCGGGCGTGCTGTGG

S9 Fig. (continued)

DENV3

GGCACAGGCAGTCAAGGCGAAACTCTGGGTGAAAAGTGGAAGAAACGTTTAAACCAGCTTTCACGCAAGGAGTTCGACTTATACAAGAAAAG
CGGAATTACCGAAGTGGACCGCACCGAGGCCAAAGAAGGTCTGAAACGTGGGGAGATTACCCATCATGCTGTTTCCCGTGGCTCGGCAAAAC
TGCAGTGGTTCGTAGAACGCAATATGGTGATCCCTGAAGGCCGCGTTATCGACCTGGGCTGTGGTCGCGGCGGTTGGAGTTATTATTGCGCC
GGGCTTAAGAAAGTTACTGAAGTACGTGGTTACACTAAAGGAGGACCGGGCCACGAAGAACCAGTACCGATGAGTACCTACGGCTGGAATAT
CGTCAAGCTGATGTCGGGGAAAGACGTGTTTTATCTGCCACCGGAGAAATGCGACACCCTTCTGTGCGATATTGGTGAATCCTCGCCAAGTC
CTACCGTGGAAGAGTCCCGTACGATTCGCGTTCTGAAAATGGTCGAACCGTGGTTGAAGAACAATCAGTTCTGTATCAAAGTGCTCAATCCG
TATATGCCGACCGTAATCGAACACTTAGAGCGCTTACAGCGCAAGCACGGTGGCATGCTGGTTCGTAACCCGCTGAGCCGTAACTCTACTCA
CGAAATGTACTGGATCAGCAATGGTACGGGGAACATCGTATCAAGCGTCAATATGGTGTCACGCCTGTTGCTGAACCGTTTTACCATGACGT
ACCGTCGCCCGACTATCGAGAAAGACGTCGATTTGGGCGCAGGCACCCGCCACGTGAACGCTGAACCAGAAACGCCAAACATGGATGTTATC
GGGGAACGCATTCGCCGCATCAAAGAAGAGCACTCCTCTACCTGGCATTACGACGATGAGAACCCGTATAAAACGTGGGCATATCATGGCTC
GTATGAAGTGAAGGCGACAGGATCCGCCTCTAGCATGATTAATGGAGTGGTGAAACTGTTAACCAAACCGTGGGATGTCGTACCGACCGTTA
CACAGATGGCGATGACCGACACTACGCCCTTTGGACAACAGCGTGTGTTTAAAGAAAAGGTCGACACTCGTACGCCCAAACCCATGCCTGGC
ACGCGCAAAGTGATGGAGATTACGGCGGGTTGGCTCTGGCGTACGCTGGGTCGTAACAAACGTCCGCGCCTGTGTACTCGCGAAGAATTTAC
TAAAAAAGTGCGCACCAATGCGGCTATGGGCGCGGTGTTTACGGAGGAAAACCAGTGGGACAGCGCACGCGCTGCAGTAGAGGACGAAGAAT
TCTGGAAACTGGTGGACCGCGAGCGTGAATTACACAAGCAAGGTAAATGCGGAAGTTGCGTCTATAATATGATGGGCAAGCGGGAAAAAAAA
CTGGGTGAATTCGGTAAAGCTAAAGGCTCTCGGGCAATTTGGTACATGTGGCTGGGTGCGCGTTATCTGGAATTTGAGGCCCTCGGTTTTCT
GAACGAAGATCATTGGTTTTCCCGCGAGAACAGCTATAGTGGTGTGGAAGGGGAAGGCCTGCATAAACTGGGCTACATTCTGCGGGATATCA
GCAAAATTCCGGGCGGGGCCATGTATGCCGATGATACAGCAGGGTGGGATACCCGCATTACCGAAGATGACTTGCACAATGAAGAGAAAATT
ACGCAGCAAATGGATCCCGAACATCGCCAGCTTGCGAACGCCATTTTCAAACTGACCTATCAGAACAAAGTTGTCAAGGTTCAGCGTCCTAC
CCCAAAGGGGACCGTTATGGATATTATCTCACGGAAAGATCAGCGGGGCTCTGGTCAGGTTGGTACCTACGGCCTTAACACATTCACGAACA
TGGAAGCCCAGTTAATTCGTCAGATGGAGGGTGAAGGTGTCTTGTCGAAAACCGACCTGGAGAACCCGCATCTCTTGGAAAAAAAAATCACC
CAGTGGCTTGAAACGAAAGGGGTGGAGCGCCTGAAACGTATGGCGATTTCGGGCGATGATTGTGTGGTCAAACCGATTGATGATCGGTTTGC
CAATGCATTGTTAGCACTGAACGATATGGGCAAAGTCCGCAAAGACATTCCGCAATGGCAACCGAGTAAAGGTTGGCATGATTGGCAGCAAG
TTCCGTTCTGCTCGCATCACTTTCACGAACTCATTATGAAAGACGGACGCAAATTGGTTGTGCCGTGTCGTCCACAGGATGAACTTATTGGC
CGTGCTCGTATTTCACAAGGCGCGGGTTGGTCACTGAAGGAAACAGCGTGTCTCGGCAAAGCCTATGCGCAAATGTGGGCGCTGATGTACTT
TCACCGCCGTGATCTCCGCCTGGCGTCTAATGCAATCTGCAGCGCCGTACCGGTTCATTGGGTGCCAACGTCGCGTACCACCTGGAGCATTC
ATGCCCATCATCAGTGGATGACTACGGAAGATATGCTGACCGTGTGGAATCGCGTGTGGATCGAAGATAATCCTTGGATGGAAGATAAAACG
CCCGTAACCACCTGGGAAGATGTTCCTTATCTGGGCAAACGCGAGGATCAATGGTGCGGGTCTTTAATCGGTCTGACCTCCCGCGCTACATG
GGCGCAGAATATCCTGACAGCGATTCAGCAAGTCCGTAGCCTGATCGGTAATGAGGAATTCCTGGATTACATGCCGAGCATGAAACGCTTTC
GCAAAGAAGAAGAGAGCGAAGGCGCGATTTGG

DENV4

TCCTTGATTAAAAATGCCCAAACACCGCGCCGTGGTACGGGCACGACCGGCGAAACGCTTGGCGAAAAGTGGAAACGCCAGTTGAACAGCCT
TGATCGCAAAGAGTTCGAGGAATATAAGCGATCAGGTATACTGGAAGTCGATCGAACTGAAGCAAAAAGTGCCCTCAAAGATGGCAGTAAAA
TCAAATATGCGGTATCCCGTGGTACAAGCAAAATTCGGTGGATTGTTGAACGCGGTATGGTTAAACCGAAAGGCAAAGTTGTGGATTTGGGG
TGTGGACGCGGTGGTTGGAGCTACTACATGGCGACTCTGAAGAATGTCACTGAGGTGAAGGGCTATACGAAAGGCGGTCCGGGCCATGAGGA
ACCCATCCCGATGGCCACCTATGGCTGGAATCTGGTGAAACTGCATTCGGGTGTTGACGTGTTCTATAAACCGACCGAACAGGTTGACACGC
TGTTGTGCGACATTGGTGAGAGCTCTTCGAATCCCACAATCGAAGAAGGTCGCACGCTGCGCGTGCTAAAAATGGTTGAACCTTGGCTTTCT
AGCAAACCGGAGTTTTGCATCAAAGTCCTGAATCCGTATATGCCTACGGTGATTGAGGAATTGGAAAAACTGCAAAGAAAACATGGCGGTAG
TCTAGTTCGCTGTCCTCTGTCTCGCAATTCAACCCACGAAATGTACTGGGTTAGCGGGGTTTCTGGTAACATTGTGTCGTCCGTCAACACTA
CCTCGAAGATGCTGCTGAATCGTTTCACGACTCGTCACCGTAAACCAACCTACGAAAAGGATGCAGATTTAGGCGCTGGCACACGCTCCGTC
TCCACCGAAACCGAAAAGCCGGACATGACGATAATTGGGAGACGTCTACAACGCTTACAAGAGGAGCACAAGGAAACCTGGCATTATGACCA
CGAAAACCCGTATCGGACTTGGGCATATCATGGGTCCTATGAAGCGCCATCAACCGGAAGTGCCAGCAGCATGGTGAACGGTGTTGTGAAAC
TCTTAACCAAACCGTGGGATGTGGTACCGATGGTGACTCAACTGGCTATGACCGATACCACACCGTTTGGTCAGCAGCGCGTGTTCAAAGAG
AAAGTCGATACGCGAACCCCTCAGCCCAAACCGGGGACTCGTGTGGTGATGACTACCACCGCGAACTGGCTGTGGGCACTGCTGGGCCGTAA
GAAAAACCCACGTTTGTGTACGCGCGAAGAATTTATCTCAAAAGTCCGCTCTAATGCAGCCATCGGTGCAGTGTTCCAGGAAGAACAAGGTT
GGACATCTGCGTCAGAGGCCGTAAATGATAGCCGCTTTTGGGAACTGGTCGATAAAGAACGCGCCCTCCATCAGGAAGGCAAATGCGAGTCG
TGCGTGTATAACATGATGGGGAAACGCGAGAAGAAACTGGGAGAGTTTGGTCGTGCGAAAGGCTCTCGTGCCATCTGGTACATGTGGCTCGG
CGCACGCTTCCTTGAATTTGAAGCGTTAGGATTTTTAAACGAAGATCACTGGTTTGGCCGTGAGAACTCGTGGTCTGGCGTAGAGGGAGAAG
GCCTGCATCGTCTGGGTTACATTCTGGAAGATATCGACAAAAAAGACGGGGACTTGATCTACGCTGATGATACCGCTGGTTGGGACACTCGT
ATCACCGAAGATGACCTGCTGAACGAAGAGCTGATCACGGAACAGATGGCGCCGCATCACAAAATTCTGGCTAAAGCGATCTTTAAGTTAAC
CTATCAGAACAAAGTGGTTAAAGTACTCCGTCCAACGCCGAAAGGAGCCGTCATGGACATTATTAGTCGGAAAGATCAACGTGGTTCTGGTC
AGGTGGGTACCTATGGCCTGAATACGTTCACGAATATGGAAGTTCAGCTGATTCGTCAGATGGAAGCGGAAGGAGTCATTACTCGTGATGAC
ATGCATAACCCAAAAGGCCTGAAAGAGCGAGTGGAGAAATGGCTGAAAGAGTGTGGAGTAGACCGCTTGAAGCGCATGGCGATCAGCGGGGA
TGATTGCGTAGTGAAGCCTCTGGACGAACGCTTTAGCACGTCGCTTCTGTTTCTGAACGATATGGGCAAAGTGCGTAAAGATATTCCCCAGT
GGGAACCTAGCAAGGGGTGGAAAAATTGGCAAGAGGTACCGTTCTGCAGCCACCATTTCCACAAGATTTTCATGAAGGATGGCCGTTCGCTG
GTGGTCCCGTGCCGCAATCAGGACGAACTCATTGGCCGCGCTCGGATTTCACAGGGTGCGGGCTGGAGCCTCCGCGAAACCGCGTGTCTGGG
CAAAGCGTACGCCCAAATGTGGTCCCTGATGTACTTTCATCGTCGGGATTTACGCCTTGCGTCAATGGCAATATGCTCCGCCGTTCCAACCG
AATGGTTTCCAACATCGAGAACCACCTGGAGTATTCATGCCCATCACCAGTGGATGACCACGGAAGATATGCTGAAAGTCTGGAACAGGGTT
TGGATCGAAGACAACCCCAATATGATCGATAAAACCCCGGTCCATAGCTGGGAAGACATTCCGTATCTCGGCAAACGTGAGGATCTTTGGTG
TGGTTCCTTGATTGGCCTGAGTAGTCGGGCGACATGGGCTAAAAACATTCAGACCGCAATCACCCAGGTGCGTAACTTAATCGGCAAAGAAG
AATACGTTGATTATATGCCGGTAATGAAACGTTACAGTGCACACTTTGAAAGCGAAGGGGTTTTA

S9 Fig. Synthesized DNA sequences of the full-length DENV1-4 NS5 proteins used in this study.
