# Supplementary material for: Discovery of a small molecule inhibitor targeting dengue virus NS5 RNA-dependent RNA polymerase
Source: PLoS Negl Trop Dis. 2019 Nov 18;13(11):e0007894. doi: 10.1371/journal.pntd.0007894 (PMC6886872; doi:10.1371/journal.pntd.0007894)
Supplement: S10 Fig — The purification processes of the recombinant DENV2 and 3 RdRp proteins are shown in the upper panels. The eluted fractions of the full-length NS5 proteins from Superdex200 are shown in the lower panels. The gels were stained with Coomassie Brilliant Blue. (PDF) [file pntd.0007894.s010.pdf]

S10 Fig.

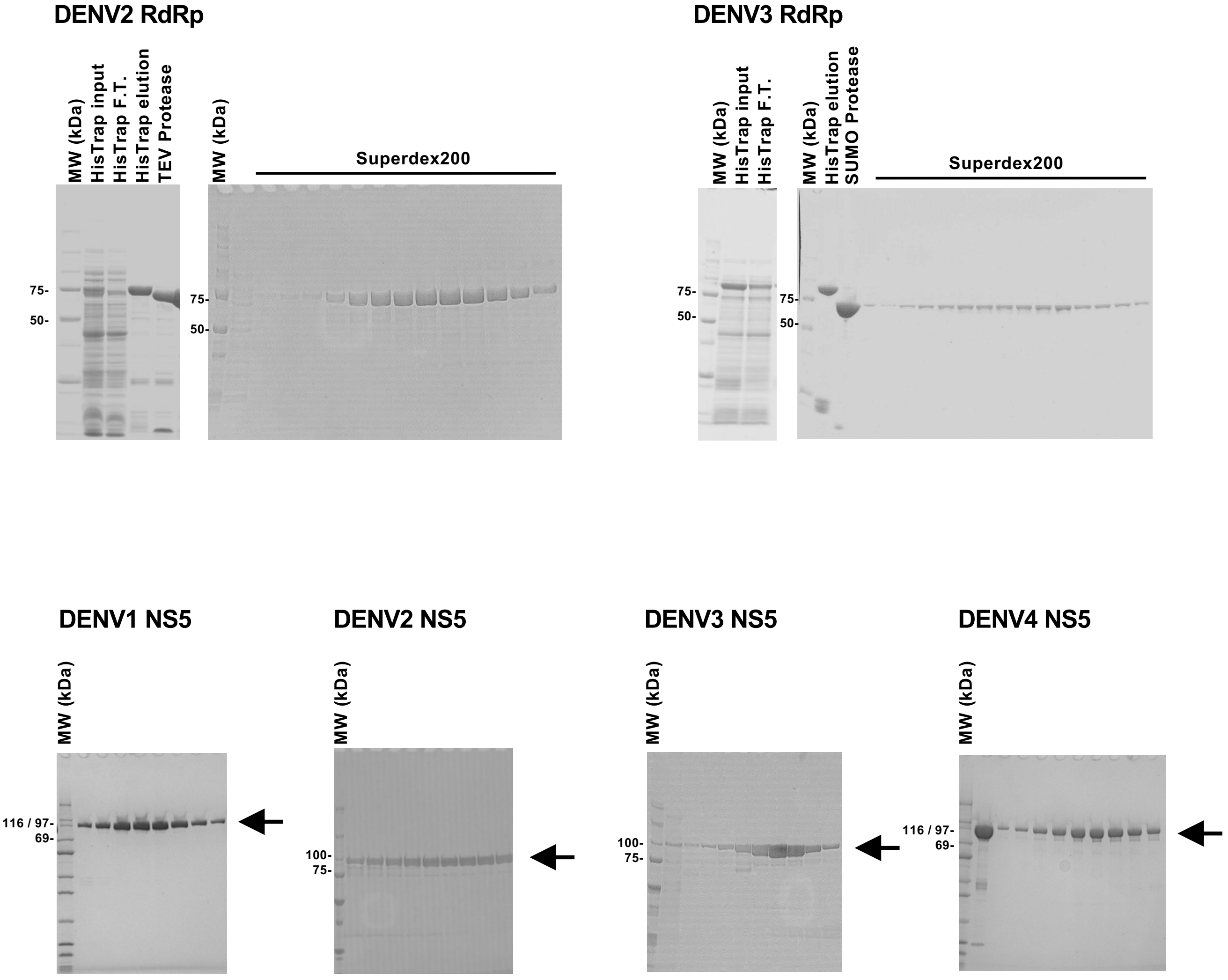

S10 Fig. SDS-PAGE analyses of the purified recombinant RdRp proteins and full-length NS5 proteins. The purification processes of the recombinant DENV2 and 3 RdRp proteins are shown in the upper panels. The eluted fractions of the full-length NS5 proteins from Superdex200 are shown in the lower panels. The gels were stained with Coomassie Brilliant Blue.
