# Supplementary material for: Discovery of a small molecule inhibitor targeting dengue virus NS5 RNA-dependent RNA polymerase
Source: PLoS Negl Trop Dis. 2019 Nov 18;13(11):e0007894. doi: 10.1371/journal.pntd.0007894 (PMC6886872; doi:10.1371/journal.pntd.0007894)
Supplement: S1 Table — (PDF) [file pntd.0007894.s011.pdf]

S1 Table

S1 Table. Data collection and refinement statistics

	DENV2 RK-0404678	DENV2 Apo	DENV3 RK-0404678	DENV3 Apo
**Data collection**				
Space group	*I*432	*I*432	$C222_1$	$C222_1$
Unit-cell (Å)	236.5, 236.5, 236.5	237.4, 237.4, 237.4	164.0, 179.3, 58.3	161.5, 177.4, 58.0
Resolution (Å)	77.2–2.43 (2.49–2.43)	53.1-2.11 (2.16-2.11)	64.7-1.97 (2.02-1.97)	62.7-2.14 (2.20-2.14)
R_{sym} (%)	20.5 (228.5)	5.8 (148.7)	8.9 (226.4)	13.3 (154.1)
$I/\sigma(I)$	16.4 (1.8)	28.2 (1.2)	13.8 (0.8)	12.1 (1.5)
Completeness (%)	100 (100)	95.7 (70.1)	99.4 (99.9)	99.9 (99.9)
Redundancy	22.3 (22.6)	13.7 (5.0)	7.3 (7.5)	7.4 (7.5)
$CC_{1/2}$	0.998 (0.601)	0.999 (0.398)	0.998 (0.609)	0.998 (0.616)
**Refinement**				
Resolution (Å)	55.7-2.43 (2.49-2.43)	42.0-2.11 (2.16-2.11)	56.2-1.97 (2.00-1.97)	51.5-2.14 (2.19-2.14)
No. reflections	42496 (2666)	62325 (3117)	60752 (2604)	46375 (3101)
R_{work} / R_{free} (%)	19.7 / 23.1 (28.9 / 30.8)	19.5 / 22.1 (30.7 / 35.3)	22.3 / 25.5 (37.5 / 37.9)	21.9 / 25.0 (32.1 / 36.0)
No. atoms Protein / Water	4769 / 207	4781 / 222	4638 / 331	4687 / 227
RMS Bonds (Å) / Angles(°)	0.008 / 0.899	0.005 / 0.746	0.005 / 0.725	0.003 / 0.518
Ramachandran Favored / Outliers (%)	97.0 / 0.35	96.5 / 0.35	96.3 / 0.18	95.6 / 0.0
