# Supplementary material for: Discovery of a small molecule inhibitor targeting dengue virus NS5 RNA-dependent RNA polymerase
Source: PLoS Negl Trop Dis. 2019 Nov 18;13(11):e0007894. doi: 10.1371/journal.pntd.0007894 (PMC6886872; doi:10.1371/journal.pntd.0007894)
Supplement: S2 Table — (PDF) [file pntd.0007894.s012.pdf]

S2 Table

S2 Table. List of primers used in this study

For the recombinant proteins	
	Sequence (5′→ 3′)
DENV1_NS5	F: CTCACAGAGAACAGATTGGTGGAGGTACCGGTGCCCAAGGGGAA<br>R: GGGCGGGGATCAATCAATCATTACCACAGCGCGCCTTCGGGATC
DENV3_NS5	F: CTCACAGAGAACAGATTGGTGGAGGCACAGGCAGTCAAGG<br>R: GGGCGGGGATCAATCAATCATTACCAAATCGCGCCTTCGCT
DENV4_NS5	F: CTCACAGAGAACAGATTGGTGGATCCTTGATTAAAAATGCCCAAAC<br>R: GGGCGGGGATCAATCAATCATTATAAAACCCCTTCGCTTTCA
DENV2_RdRp	F: CCAGCGGCTCCTCGGGAACTTACGAGCCAGATGT<br>R: CCAGCGGCTCCTCGGGAACTTACGAGCCAGATGT
DENV3_RdRp	F: GAACAGATTGGTGGAACTATCGAGAAAGACGTC<br>R: GATCAATCAATCATTATTCGCTCTCTTCTTCTTTGC
DENV1_C708A	F: GTTCCGTTTgctAGTCACCATTTCCACCAGCTGATCATG<br>R: AATGGTGACTagcAAACGGAACCTGCTGCCAATCGTTCCAT
DENV1_C708Q	F: GTTCCGTTTcagAGTCACCATTTCCACCAGCTGATCATG<br>R: AATGGTGACTctgAAACGGAACCTGCTGCCAATCGTTCCAT
DENV1_C779A	F: AACGCCATTgctAGTGCGGTGCCCGTGGATTGGGTTCCT<br>R: GCACCGCACTagcAATGGCGTTCGCTGCCAGACGTAAAT
DENV1_C779Q	F: AACGCCATTcagAGTGCGGTGCCCGTGGATTGGGTTCCT<br>R: GCACCGCACTctgAATGGCGTTCGCTGCCAGACGTAAAT
DENV2_C709A	F: GTGCCGTTTgcgAGCCATCATTTTCATGAACTGATTATG<br>R: AATGATGGCTcgcAAACGGCACCTGGGTCCAATCGTTCC
DENV2_C709Q	F: GTGCCGTTTcagAGCCATCATTTTCATGAACTGATTATG<br>R: AATGATGGCTctgAAACGGCACCTGGGTCCAATCGTTCC
DENV2_C780A	F: AACGCGATTgcgAGCGCGGTGCCGAGCCATTGGGTGCCGA<br>R: GCACCGCGCTcgcAATCGCGTTTGCTGCCAGACGCAGAT
DENV2_C780Q	F: AACGCGATTcagAGCGCGGTGCCGAGCCATTGGGTGCCGA<br>R: GCACCGCGCTctgAATCGCGTTTGCTGCCAGACGCAGAT

S2 Table (continued)

For the recombinant viruses	
	Sequence (5′→ 3′)
Fragment #1	1F: AAGCAGAGCTGGTTTAGTGAACCGGAGTTGTTAGTCTACGTGGAC<br>1R: TTTCTGTCACAATTGGGTTGACTGT
Fragment #2	2F: ACAGTCAACCCAATTGTGACAGAAA<br>2R: AGTCACTGCCAATTGATACTTTTCC
Fragment #3	3F: GGAAAAGTATCAATTGGCAGTGACT<br>3R: TCTTCCTATTCTCCCTCTTCTTTGT
Fragment #4	4F: ACAAAGAAGAGGGAGAATAGGAAGA<br>4R: ACTGAGGGCATATATGGGTTGAGAA
Fragment #5	5F: TTCTCAACCCATATATGCCCTCAGT<br>5R: GCTTCCATATTGGTGAAAGTATTGA
Fragment #6	6F: TCAATACTTTCACCAATATGGAAGC<br>6R: GAGGTGGAGATGCCATGCCGACCCTAGAACCTGTTGATTCAACAG
NS5 C709A	C704A-F: GTGCCCTTCgctTCACACCATTTCCATGA<br>C704A-R: ATGGTGTGAagcGAAGGGCACTTGTGTCC
NS5 C709Q	C704Q-F: GTGCCCTTCcaaTCACACCATTTCCATGA<br>C704Q-R: ATGGTGTGAttgGAAGGGCACTTGTGTCC
NS5 C780A	C775A-F: AATGCTATTgccTCGGCAGTACCATCACA<br>C775A-R: TACTGCCGAggcAATAGCATTTGCCGCCA
NS5 C780Q	C775Q-F: AATGCTATTcaaTCGGCAGTACCATCACA<br>C775Q-R: TACTGCCGAttgAATAGCATTTGCCGCCA

The codons of each point mutation are represented by letters in lowercase.
